# Metazoan parasite infracommunities of the dusky flounder (*Syacium papillosum*) as bioindicators of environmental conditions in the continental shelf of the Yucatan Peninsula, Mexico

**DOI:** 10.1186/s13071-019-3524-6

**Published:** 2019-05-31

**Authors:** Víctor M. Vidal-Martínez, Iván Velázquez-Abunader, Oscar Arturo Centeno-Chalé, Ana Luisa May-Tec, Lilia C. Soler-Jiménez, Daniel Pech, Ismael Mariño-Tapia, Cecilia Enriquez, Omar Zapata-Pérez, Jorge Herrera-Silveira, David I. Hernández-Mena, Sharon Z. Herzka, Uriel Ordoñez-López, M. Leopoldina Aguirre-Macedo

**Affiliations:** 1 Centro de Investigación y de Estudios Avanzados del Instituto Politécnico Nacional, Unidad Mérida, Km 6 Carretera Antigua a Progreso, Cordemex, 97310 Mérida, Yucatán México; 2 Biodiversidad Marina y Cambio Climático, El Colegio de la Frontera Sur, Av. Rancho Polígono 2-A, Ciudad Industrial, 24500 Lerma Campeche, Campeche México; 3 Facultad de Ciencias/ENES-Mérida, Universidad Nacional Autónoma de México, Puerto de abrigo s/n, 97356 Sisal, Yucatán Mexico; 4 Departamento de Oceanografía Biológica, Centro de Investigación Científica y de Educación Superior de Ensenada, Carretera Ensenada, Tijuana No., Ensenada, B.C. México

**Keywords:** Dusky flounder, Metazoan parasites, Bioindicators, Environmental impact, Hydrocarbons, Heavy metals, Contamination, Yucatan, Mexico

## Abstract

**Background:**

We assessed metrics of the metazoan parasite infracommunities of the dusky flounder (*Syacium papillosum*) as indicators of aquatic environmental health of the Yucatan Shelf (YS) prior to oil extraction. We sampled the dusky flounder and its parasites along the YS, mostly during the 2015 north wind season (November–April). Our aims were: (i) to determine whether the parasite infracommunity metrics of *S. papillosum* exhibit significant differences among YS subregions; (ii) to determine whether the probability of the occurrence of its parasite species and individuals were affected by environmental variables, nutrients, heavy metals and hydrocarbons at the seascape level; and (iii) to determine whether there were statistical differences between the parasite infracommunity metrics of *S. papillosum* from YS and those of *Syacium gunteri* from the Campeche Sound. Multivariate statistical analyses and generalised additive models (GAMs) were used to examine the potential statistical associations between the contaminants, environmental variables and parasite community metrics, and the maximum entropy algorithm (MaxEnt) was used to characterise the habitat’s suitability for the parasite’s probability of occurrence.

**Results:**

We recovered 48 metazoan parasite species from 127 *S. papillosum*, with larval cestodes and digeneans being the most numerically-dominant. Multivariate analyses showed significant differences in parasite infracommunity metrics among Western YS, Mid YS and Caribbean subregions, with the latter being the richest in species but not in individuals. The GAM and MaxEnt results indicated a negative effect of top predators (e.g. sharks and rays) removal on parasite metrics. The parasite infracommunities of *S. papillosum* were twice as rich in the number of species and individuals as those reported for *S. gunteri* from the Campeche Sound.

**Conclusions:**

The significant differences among subregions in parasite metrics were apparently due to the interruption of the Yucatan current during the north wind season. The fishing of top predators in combination with an influx of nutrients and hydrocarbons in low concentrations coincides with an increase in larval cestodes and digeneans in *S. papillosum*. The dusky flounder inhabits a region (YS) with a larger number of metazoan parasite species compared with those available for *S. gunteri* in the Campeche Sound, suggesting better environmental conditions for transmission in the YS.

**Electronic supplementary material:**

The online version of this article (10.1186/s13071-019-3524-6) contains supplementary material, which is available to authorized users.

## Background

Due to their complex life-cycles and free-living stages, metazoan parasites (hereafter called ‘parasites’) are just as exposed to anthropogenic environmental impacts (e.g. uncontrolled sewage release, contaminants, fisheries-targeting host species, etc.) in marine ecosystems as free-living organisms. In the absence of an environmental impact, it is reasonable to expect healthy ecosystems in which parasites can safely complete their life-cycles and yield parasite community metrics (e.g. number of species and individuals, diversity or numerical dominance) that fluctuate within a normal range for that specific environment [1–5]. In contrast, parasite communities of marine organisms exposed to environmental impacts (i.e. oil spills or intensive fishery activity) can show positive [6–11] or negative responses [7] in parasite community metrics. For example, after the Prestige oil-spill, Pérez-del-Olmo et al. [10] used functional groups of parasites with direct life-cycles (monoxenous) and several hosts (heteroxenous), reporting an increase in the number of species and individuals. The authors associated this increase to the environmental enrichment produced by the oil-spill in the northern Spain coast. The reason that these authors were able to detect such patterns was that they had data on parasite communities of the marine fish *Boops boops* in the region prior to the oil-spill. In another example, Centeno Chale et al. [7] had the opportunity to compare parasite communities of the Mexican halibut *Cyclopsetta chittendeni* immediately after an oil-spill in southern Mexico and six months later. These authors found a very low number of species and individuals in the parasite communities of the Mexican halibut immediately after the oil-spill (October 2007). However, after six months, they found a significant increase in the number of species and individuals, which was attributed to the carbon enrichment produced by the oil-spill and to the rescue effect of the fish and parasite populations from the surroundings. Again, having previous data on parasites allowed a sound comparison of the environmental quality of the marine habitat.

During the last 20 years, the Mexican oil company, PEMEX, has provided financial support for environmental studies along the continental shelf of the Gulf of Mexico, from which several studies on parasites as bioindicators of environmental impact have been published [3, 4, 7, 12, 13]. Most of these studies were focused on the Campeche Sound, which is where most of the oil-extraction activities in Mexican waters occur. However, since 2015, the Mexican government has offered investment opportunities to international private oil companies for both the exploration and extraction of hydrocarbons in shallow and deep Mexican waters [14]. One of these potential investment regions is the Yucatan shelf (YS) [15], where the Cayo Arcas offshore crude oil loading terminal (not an oil-extraction platform) is the only extant facility of PEMEX [16]. Whether or not oil-extraction activities will be developed in this region remains to be seen; however, there is a pressing need for baseline environmental information throughout the entire YS prior to the development of oil-extraction activities. These baseline data will allow us to describe the current environmental status of the YS, as described by parasite community metrics such as the number of parasite species and individuals. However, it is important to consider that the YS is not exempt of both natural seasonal oceanographic and anthropogenic forcings. For example, the Cabo Catoche upwelling is propelled by the Yucatan Current transporting sediment particles [17, 18], and most likely free-living invertebrates, fishes and larval parasites from east to west along the continental shelf of the Yucatan Peninsula. However, Reyes-Mendoza et al. [18] have shown that the upwelling becomes stopped during the north wind season (October to February), which indeed should affect the flux of organisms westward. Additionally, overfishing of large pelagic fishes (e.g. red groupers, snappers and sharks) [19–21] in the Campeche Bank also removes essential hosts for the completion of parasite life-cycles in the west corner of the Yucatan Peninsula. We were able to perform an oceanographic cruise during the 2015–2016 north wind season in the YS, which allowed us to obtain more than 40 environmental variables (e.g. concentrations of heavy metals and polyaromatic hydrocarbons, temperature, salinity and pH) from water, sediments and the parasite load of several flatfish species. To ensure a sound statistical analysis, a detailed study of the parasite communities of the most frequent and abundant flatfish species in the study area, the dusky flounder (*Syacium papillosum*) was performed. Therefore, based on these data and the differences in the flux of both nutrients and larval stages from east to west during the north wind season found by Reyes-Mendoza et al. [18], as well as overfishing in the north east corner of the Campeche Bank (near Cayo Arcas), we hypothesise that there will be differences in the species composition and relative abundance of the metazoan parasite communities of the dusky flounder between the zone near to Cabo Catoche and the Caribbean, with respect to the western zone of the Yucatecan shelf.

We also used ecological niche models (ENMs) to determine the influence of environmental variables on the probability of occurrence of the metazoan parasites of the dusky flounder at seascape scale (hectares to thousands of km^2^). Most of the extant ENMs are correlative in nature and determine whether there are statistical associations between environmental, biological and/or geographical variables and species abundances or occurrences to establish the sets of conditions under which the species can maintain viable populations [4]. Due to adverse weather (November 2015 north wind season in the Gulf of Mexico), fish sampling was much more difficult on the western side of the YS and the number of sampling stations was lower compared with those of the Mid or eastern YS. This asymmetry in the number of fishes did not allow a reliable comparison of the parasite component communities (number of parasite species and individuals per sampling site [22]) among subregions in the YS. Therefore, except the ENMs which are based on information extracted from geographically referenced sampling sites, our study was concentrated at the infracommunity level (number of parasite species and individuals per fish following [22]) since this hierarchical level would allow us to perform sound statistical comparisons between subregions in the YS.

Given the very low level of oil industrial activities in the YS restricted to the Cayo Arcas offshore crude oil loading terminal (http://apicampeche.com.mx/puertos-2/cayo-arcas/), it is reasonable to hypothesise that flatfish parasite communities in the YS would exhibit higher species richness and a larger number of individuals than those from the Campeche Sound, where long-term offshore oil-extraction activities are widespread. Even when the geographical distribution range of *S. papillosum* includes the Campeche Sound [23], we found few individuals there, probably because it prefers a bottom of calcareous material [23]. In the Campeche Sound, muddy and sandy bottoms dominated in our sampling stations, being the preferred habitat for the shoal flounder (*Syacium gunteri*) [3]. In fact, since *S. papillosum* and *S. gunteri* are sympatric and their feeding habits and parasite fauna overlap [23–25], we assume that their parasite infracommunities are comparable. Therefore, in addition to describing the metazoan parasite infracommunities of the dusky flounder in the YS, the aims of this paper were three-fold: (i) to determine whether the parasite infracommunity metrics of the dusky flounder in the YS exhibit significant differences among subregions from east to west; (ii) to determine whether the probability of the occurrence of the parasite species and individuals of the dusky flounder is affected by natural physicochemical environmental variables, nutrients, heavy metals and hydrocarbons at the seascape level in the YS; and (iii) to determine whether there are statistical differences between the parasite infracommunity metrics of the dusky flounder from YS and those of the shoal flounder from the Campeche Sound.

## Methods

### Study area and sampling procedures for sediment and water

**Fig. 1:**
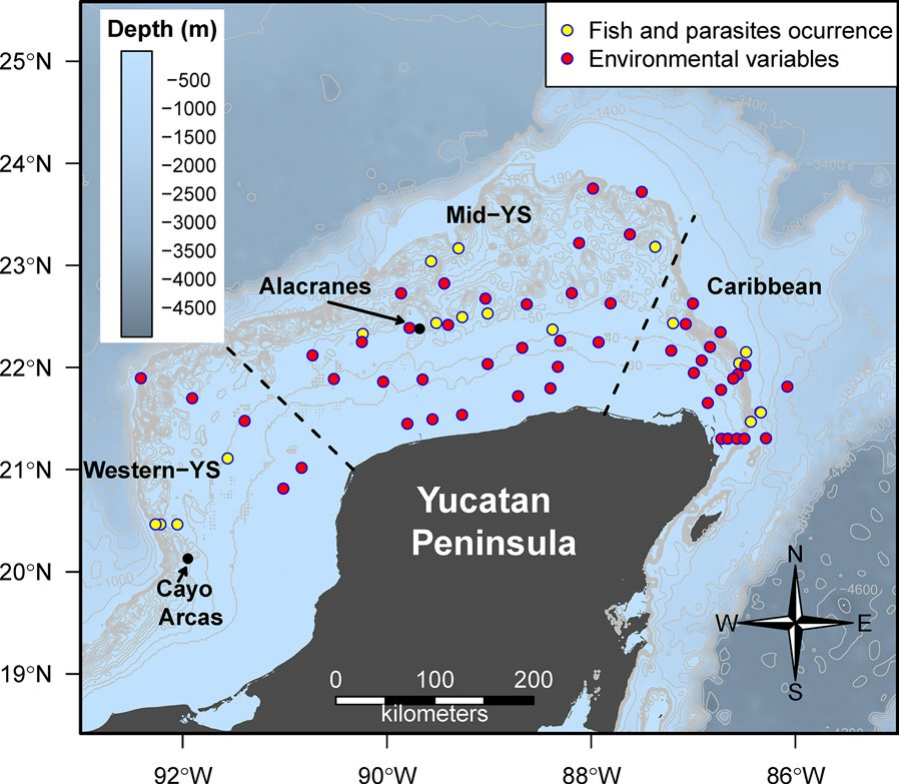
Sampling sites on the continental shelf of Yucatan, Gulf of Mexico. Yellow dots show the sampling sites where *Syacium papillosum* were caught in trawls, and red dots show the sites where water and sediment samples were obtained. Data for all environmental factors (e.g. nutrients, physicochemical variables, hydrocarbons and heavy metals) were obtained at each station

The study area along the YS included 67 sampling stations, but flatfish and their parasites were obtained only from 17 sampling stations (Fig. 1). Sediment and water samples at each of the 67 stations were obtained in November 2015 from the oceanographic vessel Riviera Maya. Benthic samples were collected using a 0.25 m^2^ Hessler Sandia MK-III box corer. Since flatfish are benthic, the water samples analysed were obtained from the bottom. These samples were obtained at depths of 15–500 m using 20 l plastic Niskin bottles set up as a rosette. These bottles were closed electronically under water to avoid contamination with surface mixtures. A total of 46 environmental variables were measured, including physicochemical variables (e.g. oxygen (mg/l), salinity (psu), pH, nitrate (µm)), hydrocarbons and heavy metals from water, sediment and organisms (Additional file 1: Table S1). Sediment samples were placed in high-density polythene bags and maintained at 4 °C for transportation to CINVESTAV-IPN-Mérida. Hydrocarbon sampling procedures are described elsewhere [3]. The physicochemical characteristics and the hydrocarbon and heavy metal concentrations of the sediment were determined at the Laboratory of Geochemistry (CINVESTAV-IPN-Mérida).

### Sampling procedures for flatfishes and helminth parasites

Due to adverse weather, fish sampling at three of the Western YS stations was performed in November 2015 from a shrimp fishing boat. Similar procedures were performed from a shrimp fishing boat during April 2016 for the remaining 14 sampling sites (Fig. 1). In the Gulf of Mexico, April is still a month when weather conditions are considered bad for navigation because the north wind season (October to February) has just finished and its influence is still present in the benthic realm. For this reason, even when our oceanographic cruise was interrupted by bad weather in November 2015, we consider that our sampling programme occurred during the north wind season of this year. Fish were collected in trawls of 0.5 h using two 20 m long shrimp nets at a speed of 2.0–2.3 knots or a distance of one nautical mile (whichever occurred first). The collected dusky flounders were placed on a board, and the total length, standard length, maximum height (cm ± 0.1 cm) and weight (g ± 0.1 g) were recorded for each individual. Each individual was subsequently examined externally for gross pathologies (e.g. tumours, ulcers, rotten fins among others etc.) before undergoing dissection to obtain samples of the gills, spleen, liver, kidney and muscle for histology (Aquatic Pathology Laboratory) and pollutant analysis (heavy metals and hydrocarbons). The remaining tissues of each individual were tagged, maintained in isolated plastic bags at − 20 °C on board the vessel and transported to the CINVESTAV-IPN Mérida Unit for parasitological examination. The *S. papillosum* collected were identified by ichthyologists at the Necton Laboratory (CINVESTAV-IPN-Mérida). The body surface, cavities and internal organs were individually examined for parasites using a dissection microscope following the procedures of Vidal-Martinez et al. [26]. All metazoan parasites from each individual host were counted in situ and preserved in 4% formalin or 96% alcohol (for monogeneans, digeneans, cestodes, nematodes and parasitic crustaceans) in labelled vials for subsequent morphological or molecular taxonomic identification. Digeneans, cestodes and acanthocephalans were stained using Mayer’s paracarmine technique, and nematodes were cleared using increasing concentrations of glycerine [26] for morphological identification. Voucher specimens were deposited in the National Helminthological Collection, Universidad Nacional Autónoma de México (CNHE) and the Helminthological Collection of CINVESTAV–IPN-Mérida. The prevalence and mean abundance of each parasite species was determined following Bush et al. [27]. Host-specialist metazoan parasite species were those previously reported in fish species of the same genus or family, whereas generalist species were those previously reported in fish species from different families.

### Data analysis

#### Infracommunity description and multivariate analyses

The infracommunity was defined as all metazoan parasites infecting an individual fish. At the infracommunity level, the mean ± SD (standard deviation) of the number of species and individual metazoan parasites per fish were calculated. Brillouin’s diversity index [22, 28] was calculated for all infracommunities and expressed as the mean ± SD per sampling station. The Brillouin’s eveness index [28] was calculated at the infracommunity level for parasites of each individual dusky flounder and presented as the mean ± SD of the index for each sampling station.

Due to their geographical proximity, all of the individual fish obtained were grouped *a priori* in three subregions: Western YS (sampling sites A3, A4, A5 and B8), Mid YS (sampling sites F29, G34, G35, H39, H40, I43 and J48) and Caribbean (sampling sites L59, O73, O75, P78, P79 and P80) (Additional file 1: Table S1). Raw quantitative infracommunity data (the number of individuals of each parasite species in/on each individual host) from each sampling station were transformed by fourth square root. A Bray–Curtis similarity matrix was built to test the hypothesis of no differences in the infracommunity composition among subregions using analysis of similarities (ANOSIM). Resemblance patterns among subregions were studied using non-metric multidimensional scaling (NMDS). SIMPER analysis was also used to determine which species contributed the most to the observed patterns of infracommunity similarity. All statistical analyses were performed following those of Pérez-del-Olmo et al. [10], but with PRIMER v.7 software.

To determine the heterogeneity of the samples obtained for each region, we used the rarefaction curves, which are plots that represent the accumulation of parasite species present in a sample as a function of the number of units sampled (number of fish) [29]. This allowed us to determine the species richness for each subregion within the data (interpolation) as well as beyond the maximum number of samples considered (extrapolation) [30]. The rarefaction curves were made with the *iNEXT* package [30] of the R programming language [31]. The confidence intervals of each curve were estimated by bootstrap procedures. The advantage of this package is that its analysis considers the Hill numbers that are known for robustness in the construction of rarefaction curves because they mathematically unify a great variety of diversity indices (differentiated by an exponent *q*) that considers both relative abundance and species richness [30, 32]. For the present study, the species richness was used as an analysis variable, considering a value of *q* = 0 for the three regions studied (more details in [30]).

#### Generalised additive models for location scale and shape (GAMLSS)

GAMLSS [33] were used to determine the statistical relationship between the environmental variables and the number of parasite species and individuals in *S. papillosum*. The multicollinearity of the predictive variables was evaluated through a variance inflation factor index, for which the *usdm* package [34] of R was used [31]. To decide which variables were discarded, a threshold of *r* = 0.65 was established (e.g. all variables with *r* < 0.65 were considered in the GAMLSS analysis).

The GAMLSS models were fitted assuming a Gumbel distribution for the number of parasite species using an inverse link function (ƞ = 1/μ), while modelling of the number of individual parasites was performed assuming a Gamma distribution with an inverse link function (ƞ = 1/μ). The choice of distribution was made based on the lowest Akaike information criterion (AIC) value in the model settings. The *gamlss* package [33] in R [31] was used to fit the models.

The best statistical model was selected by performing a forward procedure using the stepGAIC function in the *gamlss* package [33], as this assesses the contribution of each variable and their combinations in the final model through an iterative process. This function chooses the best model based on the lowest AIC value. In addition, the power of the fit of each model was evaluated through the explained deviance (ED) expressed as a percentage.

#### MaxEnt model

MaxEnt estimates the probability of a suitable habitat (expressed as probabilities from 0 to 1) establishing viable populations by finding a probability distribution that is close to being uniform but constrained by the mean values of environmental variables at the sampling stations [35]. Merow et al. [36] stated that using only the auto-features of MaxEnt could produce misleading results. Their recommendations for choosing the MaxEnt settings were followed in this study. All sampling points without the presence of *S. papillosum* and parasites were used as background data in the MaxEnt model. The number of sampling sites is critical for the performance of the model, and the size of the selected region should be biologically sound [37]. In this study, the biological justification for including all sampling sites with both the presence and absence of fish and parasites was that the presence of *S. papillosum* had been previously reported at depths of 10–140 m [38]. Therefore, there is no reason to expect that this species would be found in waters deeper than 110 m, but it is highly probable to find it between the YS and the coastal zone (Fig. 1). Therefore, each relevant environmental variable (as indicated by the percentage of ED) was interpolated to build layers that were used to predict the probability of occurrence of the number of parasite species and individuals from the models fitted. The interpolation was performed with ordinary kriging using the *automap* package [39] in R [31].

To avoid an effect of multicollinearity in the construction of the MaxEnt models, those variables used as an input for the GAMLSS models were used. In addition, a PCA among predictive layers (environmental variables) was performed to obtain the components that would explain the largest amount of variance to improve the predictions and simplify the MaxEnt model. A MaxEnt model was subsequently fitted considering the scores of the first four main axes of the PCA analysis using the iPCAProjection function of the *ENMGadgets* [40] package in R [31].

Performance of the MaxEnt models (with raw and PCA data) was evaluated using the AUC (also known as receiver operating characteristic (ROC) curves) and partial ROC (pROC) curves. The AUC method measures the capacity of a model to determine when a species is present or absent [41]. A detailed explanation of the advantages and disadvantages of the AUC and pROC curves can be found in the works of Hosmer [42], Walter [43], Phillips et al. [35], Barve [44], Peterson et al. [45], Pepe et al. [46], Liu et al. [47], Robin [48] and Peterson [49]. The R package *dismo* was used to determine the performance of the MaxEnt models [50] and produce its own pROC curves, as suggested by Pepe et al. [46]. The relevance of each model was evaluated by obtaining 20% of the presence data and 1000 random pseudo-absences to perform a cross-validation test [50].

#### Comparison of metrics of the parasite infracommunities of flatfishes from the Yucatan Shelf and the Campeche Sound

For the comparison of parasite community metrics, raw quantitative infracommunity data (the number of individuals of each parasite species in/on each individual host) of all 127 individual dusky flounders from each of the 17 sampling stations from the Yucatan shelf, and the 200 shoal flounders from 33 sampling stations of the Campeche Sound, were used [3]. All these data were transformed by fourth square root, and a Bray-Curtis similarity matrix was built to test the hypothesis of non-differences in the infracommunity composition among both regions (The Yucatan Shelf and the Campeche Sound) using analysis of similarity (ANOSIM). Resemblance patterns among both regions were studied using non-metric multidimensional scaling (NMDS). SIMPER analysis was also used to determine which species contributed the most to the observed patterns of infracommunity composition for each region. The statistical associations between the number of individuals of each parasite species and the environmental variables (Additional file 1: Table S1 in the present paper and Table S1 in [3]) obtained for each sampling point (including both regions) were analysed using distance-based redundancy analysis (dbRDA) [51]. To this aim, environmental data were log(x + 1)-transformed and standardised to construct a similarity matrix using Euclidian distances among regions. All multivariate statistical analyses were perform using Primer-e v7/ PERMANOVA + with a 95% confidence level [52]. The significance in all statistical analyses was established at α < 0.05 unless otherwise stated.

## Results

### Metazoan parasites of the dusky flounder

**Fig. 2:**
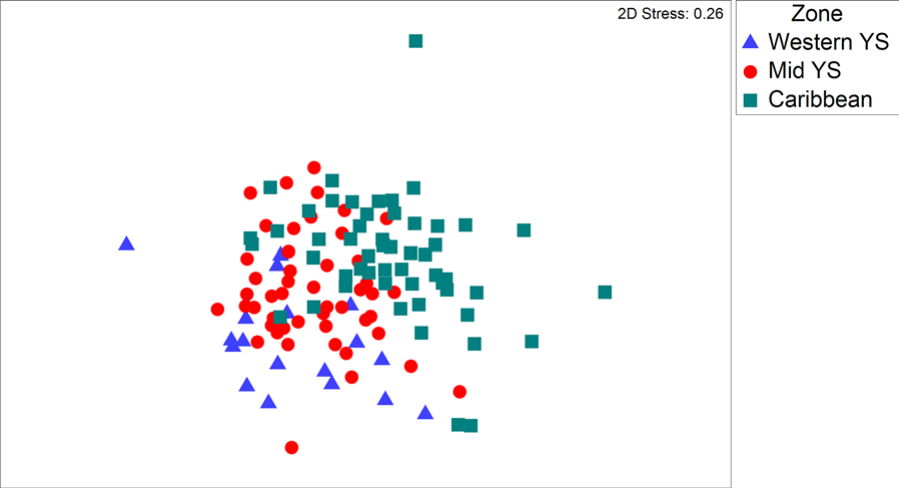
Non-metric multidimensional scaling (NMDS) of the metazoan parasites of the dusky flounder *Syacium papillosum*. The plot shows resemblance patterns from three subregions in the Yucatan Shelf

A total of 127 dusky flounders from 17 of the 67 sampling sites (Fig. 1, Table 1) were captured and examined for parasites, and 48 species and 39,571 individual parasites were recovered. The parasites infecting the dusky flounders included 1 monogenean, 21 digeneans (12 larvae and 9 adults), 8 nematodes (4 larvae and 4 adults), 10 larval-stage cestodes, 3 adult acanthocephalans, 4 adult parasitic copepods and 1 adult hirudinean (Table 2). Table 2 summarises the prevalence and mean abundance of the metazoan parasites infecting the dusky flounders at all 17 sampling stations grouped into three subregions: Western YS, Mid YS and Caribbean, as indicated by the NMDS ordination of the parasite species infracommunity composition of all 127 individual dusky flounders examined (Fig. 2). The ANOSIM pairwise test confirmed a clear separation of the Caribbean subregion from the other two subregions that showed no substantial differences between them (Table 3). In each of the three subregions, the most frequently observed and abundant species was the larval cestode *Lecanicephalum* sp., which was followed by the larval digenean *Stephanostomum* sp. 1 and the generalist adult digenean *Lecithochirium floridense*. There was no difference between male and female dusky flounders with respect to the number of parasite species (paired t-test, *t*_(0.05, 85)_ = 1.07, *P* = 0.29) or individual parasites (Wilcoxon rank sum test, *W*_(0.05, 85)_ = 997, *P* = 0.47). However, there was a significant difference in the standard length of the dusky flounders among the three subregions, with increasing fish size from the Western YS to the Caribbean subregion (ANCOVA: (*F*_(2, 116)_ = 18.55, *P* < 0.001).

**Table 1:** Morphometric data and sample size of the dusky flounder *Syacium papillosum* captured off the Peninsula of Yucatan, México

	Overall	Groups of sampling sites		
		Western YS	Mid YS	Caribbean
Total length ± SD (cm)	24.50 ± 3.22	21.76 ± 4.69	24.31 ± 3.28	25.63 ± 1.79
Standard length ± SD (cm)	21.0 ± 3.31	17.72 ± 3.90	20.74 ± 3.32	22.35 ± 2.14
Weight ± SD (g)	124.9 ± 38.60	107.66 ± 53.95	125.41 ± 43.14	129.78 ± 25.85
No. of fish examined	127	26	48	53

**Table 2:** Metazoan parasites of the dusky flounder *Syacium papillosum* off the Peninsula of Yucatan, México

Metazoan parasites	GenBank ID	CNHE or CHCM cat. number	Overall		Groups of sampling sites					
					Western YS		Mid YS		Caribbean	
			%	MA ± SD	%	MA ± SD	%	MA ± SD	%	MA ± SD
Monogenea										
*Neoheterobotrium* sp.^A, G, Ge*^	–	CHCM581	3.14	0.03	–	–	8.33	0.08	–	–
Digenea										
*Lecithochirium floridense*^A, I, Ge^	MK558793	CNHE11081	92.91	25.71 ± 84.69	88.46	58.80 ± 186.81	97.92	18.17 ± 18.98	90.57	16.32 ± 15.92
*Megalogonimus* sp.^A, I, Ge^	–	CHCM582	1.57	0.02	–	–	–	–	3.77	0.04
*Lecitochirium mecosacum*^A, I, Ge^	–	CHCM583	0.79	0.09	–	–	–	–	1.89	0.21
*Helicometrina nimia*^A, I, Ge^	MK908868	CHCM584	37	2.31 ± 7.43	–	–	27.08	2.00 ± 9.17	64.15	3.74 ± 6.75
*Zukhotrema caballeroi*^A, DT, U^	–	CHCM585	1.57	0.02	–	–	2.08	0.02	1.89	0.02
Cryptogonimidae gen. sp. 2^L, G, M, Ge^	–	CNHE11080	18.11	3.70 ± 57.45	23.07	13.15 ± 110.21	22.92	1.25 ± 7.58	11.32	1.28 ± 7.92
*Stephanostomum* sp. 1^L, A, Ge^	MK558795	CNHE11073	64.56	38.94 ± 113.94	46.15	12.00 ± 28.98	54.17	8.46 ± 26.91	83.02	79.77 ± 144.82
*Stephanostomum* sp. 2^L, G, F, Ge^	–	CNHE11074	46.45	18.08 ± 105.80	50	9.19 ± 31.57	52.08	30.54 ± 130.36	39.62	11.15 ± 102.92
*Stephanostumum* sp. 3^L, F, Ge^	–	CNHE11075	2.36	0.41 ± 16.07	–	–	–	–	5.66	1.00 ± 16.07
*Stephanostomum* sp. 4^L, G, F, Ge^	MK558796	CNHE11076	56.69	4.02 ± 18.70	26.92	0.92 ± 3.95	56.25	2.10 ± 3.30	71.7	7.28 ± 25.31
*Stephanostomum* sp. 5^L, F, M, Ge^	–	CNHE11077	28.34	13.66 ± 155.75	26.92	9.69 ± 50.74	33.33	25.75 ± 230.87	24.53	4.66 ± 26.61
*Stephanostomum* sp. 6^L, G, M, Ge^	–	CNHE11078	2.36	0.11 ± 3.21	7.69	0.26 ± 3.53	2.08	0.15	–	–
*Stephanostomum* sp. 7^L, I, U^	MK558794	CHCM586	18.11	0.76 ± 6.06	–	–	12.5	0.44 ± 3.78	32.08	1.43 ± 6.76
*Lepidapedon* sp.^A, I, Ge^	–	CHCM587	0.78	0.01	–	–	–	–	1.89	0.02
Didymozoidae (*Neotorticaecum*-like)^L, DT, Ge^	MK558797	CNHE11083	12.59	0.30 ± 3.46	7.69	0.19 ± 0.71	18.75	0.23 ± 0.44	9.43	0.43 ± 5.94
*Bulbocirrus* sp.^L, DT, U^	MK558798	CHCM617	2.52	0.09 ± 4.62	3.84	0.04	–	–	3.77	0.19 ± 5.66
*Gonocerca crassa*^A, S, Ge^	–	CHCM618	1.57	0.05 ± 3.53	–	–	–	–	3.77	0.13 ± 3.54
*Prosorhynchus* sp.^L, F, Ge^	–	CHCM588	6.29	0.24 ± 7.34	–	–	2.08	0.02	13.21	0.57 ± 7.83
*Rhiphidocotyle* sp.^L, F, Ge^	–	CNHE11084	0.78	0.01	–	–	–	–	1.89	0.02
Hemiuridae gen. sp.^A, I, U^	–	CHCM616	0.78	0.02	–	–	–	–	1.89	0.04
Cestoda										
*Lecanicephalum* sp.^L, F, U^	MK558806	CHCM589	89.9	190.84 ±246.92	88.46	76.96 ± 82.73	95.83	219.14 ± 191.10	84.91	204.87 ± 316.54
Tetraphyllidea gen. sp. ^L, I, U^	–	CHCM590	21.8	1.63 ± 8.38	23.08	1.92 ± 14.12	12.5	0.97 ± 10.08	28.3	1.85 ± 4.40
*Kotorella pronosoma*^L, DT, Ge^	MK558802	CHCM591	67.72	9.94 ± 15.58	76.92	21.84 ± 22.04	77.08	8.69 ± 9.69	54.72	5.25 ± 10.50
*Nybelinia* sp. 1^L, G, DT, Ge^	MK558803	CHCM592	23.62	0.60 ± 2.36	11.53	0.11	16.67	0.42 ± 1.69	35.85	1.02 ± 2.71
*Nybelinia* sp. 2^L, G, M, DT, Ge^	–	CHCM593	13.38	0.26 ± 1.66	23.08	0.53 ± 2.33	10.42	0.17 ± 1.34	11.32	0.23 ± 1.26
*Nybelinia* sp. 3^L, G, DT, Ge^	MK558804	CHCM594	19.68	0.54 ± 2.77	7.69	0.15 ± 1.41	20.83	0.31 ± 1.08	24.53	0.94 ± 3.41
*Nybelinia* sp. 4^L, G, Ge^	MK558805	CHCM595	22.83	0.79 ± 3.91	–	–	16.67	0.42 ± 3.07	39.62	1.53 ± 4.19
*Nybelinia* sp. 5^L, G, S, Ge^	–	CHCM596	2.36	0.14 ± 8.66	–	–	–	–	5.66	0.34 ± 8.66
*Oncomegas wageneri*^L, DT, Ge^	MK908867	CHCM597	1.57	0.02	–	–	4.17	0.04	–	–
*Lacisthorhynchus* sp.^L, Me, M, Ge^	–	CHCM598	4.72	0.10 ± 1.60	–	–	6.25	0.19 ± 2.00	5.66	0.08 ± 0.58
Pterobothridae gen. sp.^L, I, Ge^	–	CHCM599	1.57	0.06 ± 4.24	–	–	–	–	3.77	0.15 ± 4.24
Nematoda										
Anisakidae gen. sp.^L, I, Ge^	–	CHCM615	14.17	2.30 ± 16.24	15.38	2.34 ± 8.42	14.58	2.96 ± 14.71	13.21	1.70 ± 21.51
*Hysterothylacium reliquens*^L, DT, Ge^	MK558800	CHCM600	39.37	1.46 ± 4.23	50	2.07 ± 5.47	14.58	0.19 ± 0.49	56.6	2.30 ± 4.03
*Hysterothylacium fortalezae*^L, DT, Ge^	MK558799	CHCM601	14.96	0.42 ± 4.72	–	–	12.5	0.15 ± 0.41	24.53	0.89 ± 5.59
*Anisakis* sp.^L, I, Ge^	–	CHCM602	2.36	0.04 ± 1.15	3.84	0.04	4.17	0.08 ± 1.41	–	–
*Cucullanus* sp.^A, I, Ge^	–	CHCM603	22.04	0.40 ± 1.49	19.23	0.5 ± 2.07	31.25	0.60 ± 1.58	15.09	0.17 ± 0.35
*Spirocamallanus chetumalensis*^A, I, Ge^		CHCM604	24.4	1.08 ± 4.47	15.38	0.53 ± 2.64	8.33	0.19 ± 1.50	43.4	2.15 ± 4.98
*Johnstonmawsonia* sp.^A, I, Ge^	MK558801	CHCM605	4.72	0.07 ± 0.84	7.69	0.08	–	–	7.55	0.13 ± 0.96
Capillaridae gen. sp.^A, I, Ge^	–	CHCM614	0.79	0.56	3.84	2.73	–	–	–	–
Acanthocephala										
*Serrasentis sagittifer*^A, I, Ge^	MK937567	CHCM606	15.74	0.31 ± 1.19	11.53	0.35 ± 1.73	4.17	0.08 ± 1.41	28.3	0.49 ± 1.03
*Gorgorynchus lepidus*^A, I, Ge^	MK937568	CHCM607	18.11	0.67 ± 8.10	3.84	0.04	22.92	1.35 ± 11.52	20.75	0.36 ± 1.19
*Acantocephaloides plagiusae*^A, I, Ge^	–	CHCM613	0.79	0.01	–	–	–	–	1.89	0.02
Arthropoda										
*Holobomolochus confusus*^A, G, Ge^	–	CHCM608	5.11	0.08 ± 0.76	11.53	0.19 ± 1.15	22.92	0.25 ± 0.3	14.82	0.22 ± 0.76
*Acanthocandrus galerita*^A, G, U^	–	CHCM609	13.38	0.18 ± 0.61	3.84	0.07	–	–	2.08	0.02
*Gnathia* sp.^L, G, Ge^	–	CHCM610	2.36	0.03 ± 0.57	3.84	0.04	4.17	0.08	1.85	0.019
*Argulus* sp.^L, G, Ge^	–	CHCM611	0.79	0.01	–	–	–	–	3.7	0.04
Hirudinea										
*Trachellobdella lubrica*^A, G, Ge^	–	CHCM612	0.79	0.01	–	–	2.08	0.02	–	–

*Notes*: The infection parameters are prevalence (%) and mean abundance (MA ± standard deviation, SD) for 17 sampling sites (Overall) and the three groups of sampling sites formed for the NMDS analysis (see Fig. 2). ^A^adult parasite, ^L^larval parasite, ^F^fins, ^G^gills, ^I^intestine, ^Me^mesenteries, ^M^muscle, ^DT^digestive tract, ^S^stomach, ^Ge^generalist parasite, ^U^unknown status, ^*^new species to be described elsewhere. Cat. number CNHE or CHCM: Catalogue number in the National Helminthological Collection, Universidad Nacional Autónoma de México, or the Helminthological Collection of CINVESTAV-IPN-Mérida respectively

**Table 3:** Significant differences in similarity by ANOSIM for parasites of *Syacium papillosum* in Yucatan Shelf subregions

Test	*R*-statistic	*P*-value
Global for subregions	0.23	0.01
Pairwise comparisons		
Western YS *vs* Mid YS	0.19	0.26
Western YS *vs* Caribbean	0.35	0.01
Mid YS *vs* Caribbean	0.20	0.01

*Abbreviation*: ANOSIM, analysis of similarities

### Metazoan parasite infracommunities of the dusky flounder

**Fig. 3:**
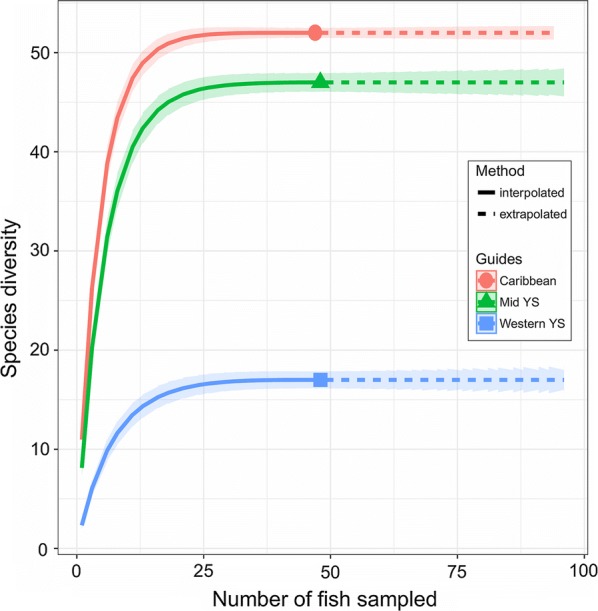
Rarefaction curves for the metazoan parasite species richness for dusky flounder *Syacium papillosum*

The metrics of the metazoan parasite infracommunities of the dusky flounder in the Western YS, Mid YS and the Caribbean subregions are presented in Table 4. Overall, there was a significant difference between subregions with regards to the number of species per fish (*F*_(2, 127)_ = 8.94, *P* < 0.0002), with a smaller number of species in Western and Mid YS than in the Caribbean subregion (Tukey’s test, α = 0.05; HSD = 1.73). Species richness R was calculated for each subregion (Fig. 3) using each individual fish as a sampling unit.

**Table 4:** The infracommunities of the metazoan parasites of the dusky flounder *Syacium papillosum* (*n* = 127)

	Overall	Western YS	Mid YS	Caribbean
Mean no. of species	8.67 ± 3.43	6.79 ± 2.74	8.29 ± 3.28	9.94 ± 3.39
Mean no. of individuals	321.12 ± 321.09	228.50 ± 186.67	334.10 ± 371.75	354.81 ± 320.24
Brillouinʼs diversity index	0.94 ± 0.44	0.96 ± 0.43	0.87 ± 0.43	0.98 ± 0.45
Brillouinʼs evenness index	0.48 ± 0.21	0.56 ± 0.22	0.44 ± 0.19	0.47 ± 0.22
Numerically dominant species	*Lecanicephalum* sp.	*Lecanicephalum* sp.	*Lecanicephalum* sp.	*Lecanicephalum* sp.

*Notes*: The data include the community metrics for 17 sampling sites (Overall) and the three groups of sampling sites formed by non-metric multidimensional scaling (NMDS) patterns from the continental shelf of the Peninsula of Yucatan in Fig. 2

However, there was no significant difference between subregions with regards to the number of individuals per fish (Kruskal-Wallis test, *H*_(2, 127)_ = 3.97, *P* = 0.14). There was no significant difference between the subregions with regards to Brillouin’s diversity index (Table 4) (*F*_(2, 124)_ = 0.97, *P* = 0.38) or Brillouin’s evenness index (*F*_(2, 124)_ = 2.48, *P* = 0.09).

The SIMPER analysis identified the species that made the largest contribution to the total number of individuals within each subregion (Table 5). At the Western YS subregion, five species contributed 90% of the total abundance. In contrast, for the Mid YS and Caribbean subregions, seven and ten species, respectively, contributed about 90% of the abundance. The larval cestode *Lecanicephalum* sp. was the most abundant parasite at the three subregions followed by the larval cestode *Kotorella pronosoma* and the generalist adult digenean *Lecithochirium floridense* at the Western YS and the Mid YS. *Lecithochirium floridense* and the larval digenean *Stephanostomum* sp. 1 were the other two species with the largest contribution to the abundance at the Caribbean subregion.

**Table 5:** Species contributing the most to the total number of individuals per subregion in SIMPER analysis

Species per subregion	Mean similarity ± SD	Percent contribution	Cumulative percent
Western YS			
*Lecanicephalum* sp.	17.72 ± 1.82	36.79	36.79
*Kotorella pronosoma*	11.39 ± 1.53	23.66	60.45
*Lecithochirium floridense*	11.34 ± 2.05	23.54	83.99
*Stephanostomum* sp. 1	1.44 ± 0.37	3.00	86.99
Cryptogonimidae gen. sp. 1	1.44 ± 0.39	2.98	89.98
Mid YS			
*Lecanicephalum* sp.	20.65 ± 2.24	40.44	40.44
*Lecithochirium floridense*	11.52 ± 2.51	22.56	62.99
*Kotorella pronosoma*	6.07 ± 1.09	11.88	74.88
*Stephanostomum* sp. 1	2.45 ± 0.60	4.80	79.67
*Stephanostomum* sp. 4	2.26 ± 0.65	4.42	84.09
*Stephanostomum* sp. 2	2.20 ± 0.55	4.31	88.4
Cryptogonimidae gen. sp. 1	1.67 ± 0.48	3.26	91.66
Caribbean			
*Lecanicephalum* sp.	12.62 ± 1.56	26.86	26.86
*Lecithochirium floridense*	8.31 ± 1.72	17.69	44.55
*Stephanostomum* sp. 1	8.07 ± 1.11	17.19	61.74
*Stephanostomum* sp. 4	4.08 ± 0.95	8.68	70.42
*Helicometrina nimia*	2.72 ± 0.78	5.78	76.2
*Kotorella pronosoma*	2.06 ± 0.62	4.38	80.58
*Hysterothylacium reliquens*	2.00 ± 0.65	4.26	84.85
*Spirocamallanus chetumalensis*	1.12 ± 0.46	2.38	87.23
*Stephanostomum* sp. 2	1.04 ± 0.40	2.20	89.43
*Nybelinia* sp. 4	0.91 ± 0.42	1.95	91.38

*Abbreviation*: SIMPER, similarity percentages; SD, standard deviation

### Statistical associations between environmental and biological variables and the number of parasite species and individuals

The statistical associations between environmental and biological variables and the number of the parasite species and individuals are presented in Table 6. Of the 46 predictive variables (Additional file 1: Table S1), only 37 did not exhibit multicollinearity and could be used to build the generalised additive models for location, scale and shape (GAMLSS). After further selection, the GAMLSS model for the number of parasite species per sampling station correlated only with fishing zone, fishing effort and nitrate concentration, which had a 44% overall contribution to the explained deviance (ED) (Table 6).

**Table 6:** Best GAMLSS models for the parasite species (NSPP) and individual numbers (NIND) of *Syacium papillosum*

Model	*df*	Global deviance	Percent of explained deviance	Akaike information criterion	*P*
NSPP ~ (ZONE) + *cs*(FE) + *cs*(NO_3_)	12	44.57	44.30	68.57	< 0.001
NIND~ *cs*(ZOOVOL) + *cs*(ASUMALI) + *cs*(SSUMPAH) + *cs*(LT) + *cs*(NOFISH) + *cs*(S235TRI) + *cs*(SANTR) + *cs*(ICTABU) + *cs*(ACEN)	41	20.73	91.13	102.73	< 0.001

*Notes*: The best GAMLSS model was selected using a stepwise procedure and the lowest values of AIC and global deviance. The independent variables (those without cs) had a linear relationship with the dependent variables

*Abbreviations*: ACEN, acenaphthylene in water; ASUMALI, sum of aliphatic hydrocarbons in water; *cs*, cubic spline smooth function; df, degrees of freedom; FE, fishing effort; ICTABU, ichthyoplankton abundance; LT, fish total length; NOFISH, number of fish per sampling station; NO_3_, nitrates concentration in water; SANTR, anthracene in sediment; SSUMPAH, sum of polyaromatic hydrocarbons in sediment; S235TRI, 2,3,5, trimethylnapthalene; ZONE, fishing zone in the Yucatan Peninsula; ZOO, zooplankton volume in water

The GAMLSS model for the number of parasites was much more complex, retaining nine (Table 6) of the 46 environmental variables measured at each sampling site (Additional file 1: Table S1). The variables retained were related to the biology of the fish (e.g. standard length, the number of fishes caught per sampling station and zooplankton and ichthyoplankton biomass) as well as several hydrocarbons (Table 6).

**Fig. 4:**
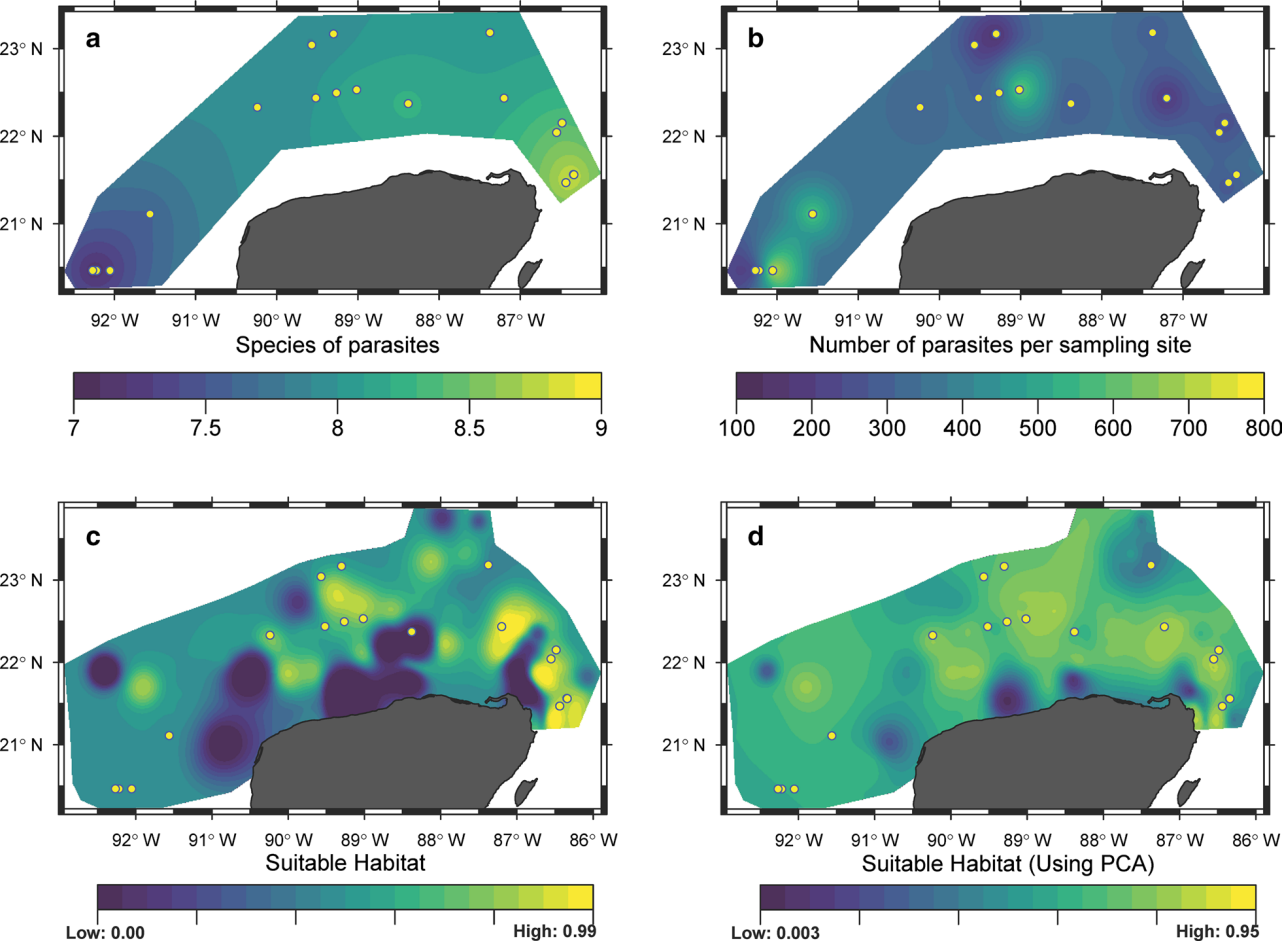
Geographical distribution of *Syacium papillosum* parasites on the Yucatan Shelf (YS). **a** Spatial representation of a GAMLSS, where the number of parasite species of *Syacium papillosum* was the dependent variable and the fishing zone, fishing effort and nitrates were independent variables. **b** Spatial representation of the GAMLSS model, where the number of parasites per sampling site was the dependent variable and zooplankton volume, sum of aliphatic hydrocarbons in water, sum of polyaromatic hydrocarbons in sediment, fish length, number of fish per sampling site, 2,3,5-trimethylnaphthalene, anthracene in sediment, ichthyoplankton abundance and acenaphthylene in water were the independent variables. **c** Suitable habitat (= probability of occurrence) for parasite individuals in *S. papillosum* along the border of the continental shelf of the YS, based on habitat suitability for raw data of 23 environmental variables (e.g. Cd, Pb, 17 HCs, SO_2_, salinity, total carbon and sediment grain size). **d** Probability of occurrence of parasite individuals in *S. papillosum* along the border of the continental shelf of the YS, based on habitat suitability for the first four principal components selected from the raw values of the 23 environmental variables in **c**. Points indicate sites at which parasites occur

The spatial representation of the number of parasite species per sampling station indicated between seven and eight species per sampling site (purple and green zones) for the Western and Mid YS subregions, and up to nine species per sampling site (yellow zone) for the Caribbean subregion (Fig. 4a). Spatial representation of the number of parasites individuals (Fig. 4b) indicated that there were between 100 and 400 parasites per sampling station in most parts of the study area (purple and blue zones) and almost 800 individuals in some hotspots (yellow zones) of the Western and Mid YS subregions.

The MaxEnt model retained only 24 of the 46 environmental variables obtained at each sampling site (Additional file 1: Table S1). The habitat suitability map, based on the probability of occurrence of the parasite species as a function of raw values of the environmental variables, is represented in Fig. 4c and suggests a high probability of occurrence of parasite species in central and eastern areas of the YS. The environmental variables retained by MaxEnt included heavy metals (e.g. Cd and Pb) and 17 hydrocarbons at low concentrations, as well as SO_2_, salinity, total organic carbon in the sediment and sediment grain size. However, this model had a relatively low area under the curve (AUC = 0.78). Predicting the probability of occurrence improved (yellow zones; AUC = 0.84) when the first four axes of the principal component analysis (PCA) were used as predictive layers (cumulative explained variance: 91.53%) (Fig. 4d).

### Comparison of metrics of the parasite infracommunities of flatfishes from the Yucatan Shelf and the Campeche Sound

**Fig. 5:**
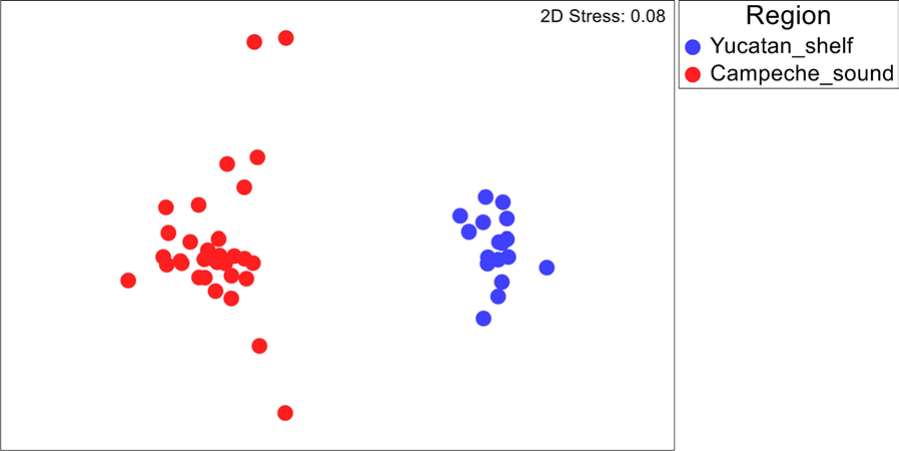
Non-metric multidimensional scaling (NMDS) of the metazoan parasites of *Syacium papillosum* and *Syacium gunteri*. The plot shows the resemblance patterns of the parasite infracommunities of *S. papillosum* for the Yucatan Shelf, and of *S. gunteri* for the Campeche Sound, southern Gulf of Mexico

There were significant differences between the infracommunities of the dusky flounder from the Yucatan Shelf and those of the shoal flounder from the Campeche Sound for both the number of species per fish (paired t-test, *t*_(0.05, 119)_ = − 22.16, *P* < 0.0001) and the number of individual parasites per fish (Wilcoxon signed-rank test, *Z*_(0.05, 119)_ = − 9.32, *P* < 0.0001). The NMDS ordination of the parasite infracommunities of all the 127 individual dusky flounders from the Yucatan shelf, and the 200 shoal flounders from the Campeche Sound, revealed a clear separation of the two regions (Fig. 5) which was confirmed by ANOSIM (*R* = 0.87, *P* = 0.0001).

The SIMPER analysis identified the species that made the largest contribution to the total number of individuals within each region (Table 7). At the Yucatan Shelf, four species contributed with 76.88% of the total abundance. In contrast, for the Campeche Sound, only one species contributed with the 78.53% of the abundance (Table 7).

**Table 7:** Species contributing the most to the total number of individuals per region in the SIMPER analysis

Species per region	Mean similarity ± SD	Percent contribution	Cumulative percent
Yucatan Shelf			
*Lecanicephalum* sp.	16.25 ± 1.78	35.66	35.66
*Lecithochirium floridense*	10.00 ± 2.00	21.95	57.61
*Kotorella pronosoma*	4.53 ± 0.85	9.93	67.54
*Stephanostomum* sp. 1	4.25 ± 0.73	9.33	76.88
Campeche Sound			
*Prochristianella hispida*	36.12 ± 1.23	78.53	78.53

**Fig. 6:**
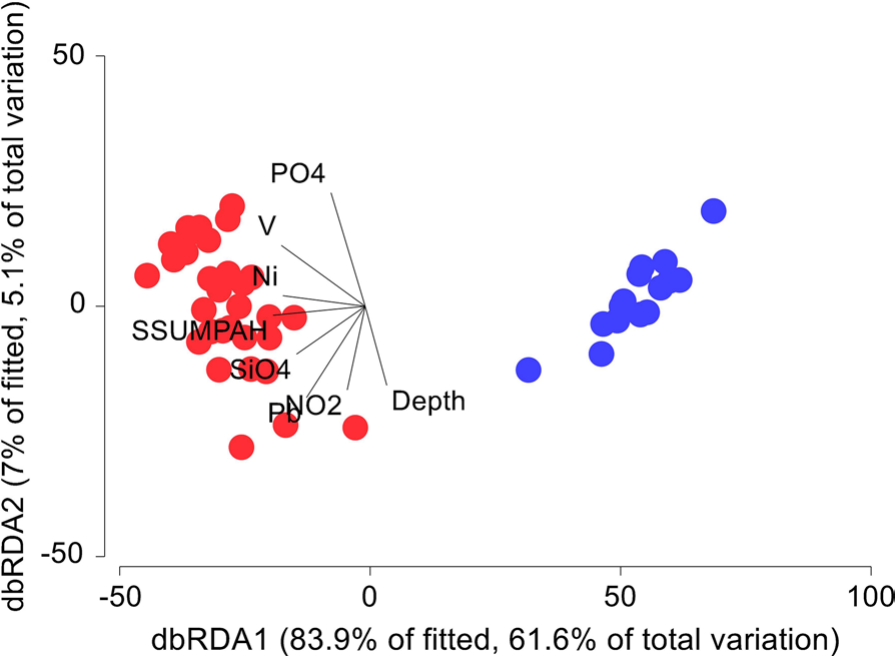
Distance-based redundancy analysis of the number of parasites of *Syacium gunteri* and *Syacium papillosum*. The plot shows the statistical associations of the number of parasites for *S. gunteri* (red dots) and *S. papillosum* (blue dots) per sampling station and the environmental variables retained by the analysis after a forward stepwise procedure. The acronyms of the environmental variables are as follows: SSUMPAH, sum of the concentrations of polyaromatic hydrocarbons per sampling site; PO_4_, phosphorus; NO_2_, nitrite; SiO_4_, silicate; Ni, nickel; Pb, lead; V, vanadium

Figure 6 shows the statistical associations between the eight environmental variables explaining the greatest amount of variance and the relative abundance of the metazoan parasite species of the infracommunities of *Syacium papillosum* for the Yucatan Shelf and *Syacium gunteri* for the Campeche Sound. The most relevant pattern in Fig. 6 was the positive association between the total number of parasites of *S. gunteri* per sampling station (red dots) and the concentrations of polyaromatic (SSUMPAH) hydrocarbons, phosphorus (PO_4_), nitrite (NO_2_), silicate (SiO_4_), heavy metals (Ni, Pb, V) and depth from the Campeche Sound. In contrast, the total number of parasites of *S. papillosum* per sampling station (blue dots) was opposed to the values of these environmental variables (Fig. 6).

## Discussion

Our results suggest that the YS is not a homogeneous region since the ecological analyses (e.g. rarefaction analysis, NMDS, ANOSIM, SIMPER, etc.) of the parasite infracommunities of the dusky flounder suggested the existence of two subregions. One subregion clearly formed by western and middle YS, which was lower in number of species compared with the Caribbean subregion. There were also significant statistical associations between environmental variables (i.e. fishing effort and hydrocarbon and heavy metal concentrations) and the probability of the occurrence of parasite species and individuals of the dusky flounder in the YS. Despite these associations, the concentrations of these contaminants in water, sediment and flatfishes were not high enough to prevent parasites from persisting and their life-cycles from being completed, as suggested by the high number of species and individuals collected. In fact, by comparing the parasite infracommunity metrics obtained for *S. papillosum* in the YS (a non-oil-extraction region) with those of *S. gunteri* from the Campeche Sound (an oil-extraction region), the former had a significantly higher number of parasite species and individuals than the latter. Therefore, based on the assumption of Hudson et al. [1] that a healthy ecosystem is that with a large number of parasite species and individuals, we suggest that the YS can be considered as such under the present environmental circumstances. In the following paragraphs, after a brief account of the life-cycles of the most frequent and abundant parasites, each of the hypotheses expressed in the introduction section has been addressed.

### Prevalence and abundance of the most frequent and abundant species

With few exceptions of new species that will be properly described elsewhere, most of the parasites recovered from the dusky flounder were all generalist autogenic parasites previously reported in marine fishes from the southern Gulf of Mexico including other flatfishes such as the tonguefish (*Symphurus plagiusa*) [53], Mexican flounder (*Cyclopsetta chittendeni*) [7], shoal flounder (*Syacium gunteri*) [3], serranids such as the red grouper (*Epinephelus morio*) [54], lutjanids such as the grey snapper (*Lutjanus griseus*) [55] and carangids such as the Florida pompano (*Trachinotus carolinus*) [56].

Of the 48 parasite species identified, 62% of individuals were larval cestodes and 36% were digeneans, with the remaining parasites representing only 2% (Table 2). The most likely explanation for the presence of these larvae throughout the study area is that they infect the dusky flounder on the sea floor of relatively healthy coral reef ecosystems (i.e. ecosystems with a low level of chemical or anthropogenic pollution) that are present throughout the YS and have enough copepods, crabs, shrimps and other small vertebrates and invertebrates acting as intermediate hosts. Also, the fact that a large proportion of species infecting the dusky flounder were in the larval stage (55%) indicates that this fish species acts as an intermediate host for many of these parasites.

Although every possible effort was made to identify the parasitological material at the species level, in several cases this was not possible (Table 2). To support our claim that these larvae belonged to specific species, we have provided morphological measurements (Additional file 2) and the molecular identification of as many parasites as possible (see Table 2). However, for several larval digeneans and cestodes, it was not possible to assign them to specific species, mainly because the adult stages have not been studied molecularly and there are no reference sequences in the GenBank database. An important point to consider is that, in contrast to regions with a long tradition in the study of parasite life-cycles such as Europe [e.g. 57–59], the Gulf of Mexico is both highly diverse in terms of the number of extant species and poorly studied with respect to the life-cycles of marine parasites. Below, we present a brief account of the intermediate and definitive hosts involved in the life-cycles and transmission of the most frequent and abundant metazoan parasites registered in *S. papillosum*. For the most frequent and abundant digeneans in the larval stage, such as *Stephanostomum* spp. and Cryptogonimidae gen. sp. (Table 2), it is likely that gastropods act as first intermediate hosts [60–62]. Predatory fishes such as serranids, lutjanids or carangids (among others) could be the definitive hosts of these digeneans (see checklist 1 in [63]). The adult digenean *Lecithochirium floridense* uses *S. papillosum* as a definitive host (Table 2) among other bony fishes [63]. The life-cycle of this species has not been described, but based on information from the relative *Hemiurus communis*, the first intermediate host can be gastropods, and marine copepods, Chaetognatha and Ctenophora can act as second intermediate hosts [64]. The tetraphyllidean cestodes use sharks and rays as definitive hosts (see checklist 1 in [65]), and the larval stages have been recorded from bivalves, gastropods and bony fishes in the Gulf of Mexico [66]. The two most frequent and abundant trypanorhynch cestodes recovered from *S. papillosum* (*K. pronosoma* and *Nybelinia* spp.) belong to the superfamily Tentacularioidea. The life-cycles of the members of this superfamily include four or more host, with copepods as first intermediate hosts, euphausiids or schooling fish as second intermediate hosts, and other fish as paratenic hosts [58]. The life-cycle of the nematode *Hysterothylacium reliquens* has also not been described. However, the life-cycle of *Hysterothylacium aduncum* includes copepods, amphipods, shrimps and isopods as first intermediate hosts, and chaetognaths and other crustaceans as second intermediate hosts. Several species of bony fish act as both paratenic hosts (that we think is the role of *S. papillosum*) and predatory fish act as definitive hosts [67, 68]. The life-cycle of *Spirocamallanus chetumalensis* has not been described. However, based on the life-cycle of a marine relative *Camallanus* (*Spirocamallanus*) *pereirai*, it is assumed that the first intermediate hosts should be copepods [69], the second ones shrimps [70], with the definitive host fishes predating on shrimps [69]. For the remaining low prevalence (less than 10%) metazoan parasites in Table 2, those with heteroxenous life-cycles (e.g. acanthocephalans, nematodes) have been most probably trophically acquired by *S. papillosum*, partially because this is a benthic predator that feeds on zoobenthic fishes and invertebrates [71], or by passive exposure to infective stages of digeneans. The exception to this pattern were those parasites with monoxenous life-cycles (monogeneans, parasitic copepods, isopods, branchiurids and leeches).

### Metazoan parasite infracommunities of the dusky flounder

The hypothesis that the parasites of the dusky flounder in the YS exhibit significant differences in similarity among subregions from east to west was accepted, as the NMDS (Fig. 2), ANOSIM (Table 3) and SIMPER (Table 5) analyses clearly showed the existence of two subregions. One subregion was formed by western and middle YS, which was significantly different from the Caribbean subregion in terms of the Bray–Curtis similarity values. Since the oceanographic sampling was performed basically during the north wind season, apparently, the transport process of sediment particles [17], and most likely free-living invertebrates, fishes and larval parasites from east to west along the continental shelf of the Yucatan Peninsula, could be interrupted, as suggested by Reyes-Mendoza et al. [18]. By using oceanographic and time series data, these authors have shown that the Cabo Catoche upwelling from east to west is stopped during the north wind season (October to February), which indeed should affect the flux of organisms westward. However, if the Yucatan Current is re-established after the north wind season, it could be expected that the flux of organisms westward would also become re-established. If this is the case, it could be expected that larval parasites would be transported by the predominantly eastward-flowing ocean currents from one sampling site to another in a process ‘seeding’ the region seasonally [72].

The mean number of parasite species per fish on the dusky flounder (Table 4) was at least twice as high as previously described for other flatfish species in the Gulf of Mexico (e.g. Mexican flounder *C. chittendeni*: 1.19 ± 1.17 to 3.27 ± 1.64 [7]; tonguefish *S. plagiusa*: 2.00 ± 0.55 to 3.00 ± 0.66 [17]). However, the diversity values for parasite infracommunities of the dusky flounder (Table 4) fall within the range observed in Campeche Sound for *C. chittendeni* (0.64 ± 0.35), *S. gunteri* (1.16 ± 0.53) and *S. plagiusa* (0.24 ± 0.11) [3, 7, 17]. The relatively low diversity values of the infracommunities of the dusky flounder and the high levels of numerical dominance within sites (Table 4) were most likely due to the large number of larval parasites. Similar low diversity and high numerical dominance at the infracommunity level was observed in the parasite communities of *C. chittendeni* [7] and *S. gunteri* [3] due to larval parasites.

With respect to rarefaction (Fig. 3), even though the number of individual fish obtained from the Caribbean and Mid YS subregions was larger than the number from Western YS, the difference in species number is unlikely to be attributable to sampling intensity, as all species accumulation curves reached their asymptotes well before the maximum number of fish per subregion was reached (Fig. 3). The most likely interpretation for the larger number of species in the Caribbean and Mid YS subregions with respect to Western YS, is that the Cabo Catoche upwelling from east to west becomes stopped during the north wind season (October to February), which indeed should affect the flux of organisms westward [18]. However, in addition to this seasonal oceanographic pattern, overfishing of large pelagic fishes (e.g. red groupers, snappers and sharks) and crustaceans (e.g. several species of shrimps) [19–21] in the Campeche Bank could also remove essential hosts for the completion of parasite life cycles in Western YS.

### Statistical associations between environmental and biological variables and the number of parasite species and individuals

Our second hypothesis was that the probability of occurrence of the parasite species and individuals of the dusky flounder would be affected by natural physicochemical environmental variables, nutrients and polycyclic aromatic hydrocarbons at the seascape level in the YS. This hypothesis was accepted since the GAMLSS model showed that the fishing zone, fishing effort and nitrate concentration had an effect on the ED (44%) of the number of parasite species (Table 6). In the yellow zone (Caribbean), higher nitrate concentrations and lower fishing pressures on potential intermediate host (e.g. crustaceans and non-carnivorous fishes) were apparently associated with large numbers of parasite species in *S. papillosum* (Fig. 4a). Conversely, in Western and Mid YS, higher fishing pressures, lower nitrate concentrations, and the seasonal interruption of the Cabo Catoche upwelling may negatively affect the number of parasite species in flatfishes. Unfortunately, the information obtained during this study is not enough to determine whether the proximity of the Cayo Arcas sampling sites to the PEMEX facility there affects the number of parasite species and individuals; a specific sampling design will be necessary to answer this question. Fishing negatively affects the life-cycles of parasites by removing top predators (e.g. sharks, rays and large pelagic fish), which are definitive hosts of their adult (i.e. reproductive) stages. Similar negative effects of fishing on parasite species were reported by Sasal et al. [73], Bartoli et al. [74] and Marzoug et al. [8] for the Mediterranean Sea, by Lafferty et al. [6] for Kiritimati Island and by Wood et al. [9] for the Line Islands in the Equatorial Pacific for coral reef fishes. In addition, Wood et al. [75] also reported the positive effects of productivity on the number of parasite species and individuals.

With regards to the number of parasite individuals, the relevant variables of the GAMLSS model were the standard fish length and abundance, zooplankton and ichthyoplankton abundance and five hydrocarbons (Table 6), thus suggesting that parasite load is related to the biology of the fishes as well as to their secondary productivity. In the yellow and green zones in Fig. 4b, there are populations of sharks and rays that benefit from the elevated nutrients and low levels of hydrocarbons and heavy metals provided by an east-to-west detachment of the upwelling off Cabo Catoche [76]. With an increase in the number of definitive hosts, there is also an increase in the number of eggs and larval stages of parasites, such as larval cestodes using *S. papillosum* as an intermediate host [77]. Therefore, in the yellow and green zones of Fig. 4b, the most likely interpretation is that parasites, such as the larval cestodes *Lecanicephalum* sp., the larval digenean *Stephanostomum* spp., the adult digenean *L. floridense* and a probable long list of other rare species in Table 1, increased in response to productivity. Conversely, in the blue zones in Fig. 4b, the removal of sharks and rays combined with fast oceanic currents in the Yucatan channel probably over-dispersed the parasite larval stages and affected the completion of their life-cycles, thus resulting in a low number of individual parasites. Lafferty et al. [6], Marzoug et al. [8] and Wood et al. [9, 75] reported that those cestodes using sharks as definitive hosts could be at risk due to overfishing. In this study, flatfishes were also definitive hosts for some parasites (e.g. *L. floridense*) and intermediate hosts for others (i.e. larval cestodes and digeneans). This suggests that, given the selective removal of top predators (sharks and rays) in specific fishing zones, there may be a higher density of *S. papillosum* due to decreased predation, which in turn could explain the higher number of adult digeneans as well as the larval cestodes and digeneans using *S. papillosum* as second intermediate host.

Similar to the results obtained with the GAMLSS models, the MaxEnt models using raw variables or PCA components (Fig. 4c and d) suggested that the habitat suitability for the occurrence of parasite species was good in the yellow and green zones and bad in the blue zones along the YS. The environmental variables selected by the models included heavy metals (e.g. Cd and Pb) and 17 hydrocarbons (all of which were found in low concentrations), SO_2_, salinity, total carbon and sediment grain size (Additional file 1: Table S1). The general interpretation of these results is similar to that for the GAMLSS models. Whether or not the fishing-mediated removal of hosts affects the life-cycles of parasites, it is clear that there are more species and individual parasites in the YS compared to other regions in the Gulf of Mexico [3, 7, 53].

### Comparison of the parasite infracommunity metrics of flatfishes from the Yucatan Shelf and the Campeche Sound

Our third hypothesis was that the parasite infracommunities of the dusky flounder in the YS would exhibit higher species richness and a larger number of individuals than those of the shoal flounder from the Campeche Sound. The results suggest that this was the case, since the NMDS (Fig. 5), SIMPER (Table 7) and distance-based redundancy analysis (Fig. 6) analyses showed the presence of two groups of sampling sites, one belonging to the YS, and the other one to the Campeche Sound. In the SIMPER analysis, the number of species contributing to the overall number of the metazoan parasite individuals was higher in the YS, compared with the Campeche Sound (Table 7). These results suggest that dusky flounders inhabit a region with a larger number of metazoan parasite species in the YS, compared with those that are available for *S. gunteri* in the Campeche Sound. Such a difference supports the hypothesis that the environmental conditions in the YS provide better opportunities for parasites to complete their life-cycles than in the Campeche Sound. The results of the distance-based redundancy analysis showed that the number of parasite species and individuals infecting *S. gunteri* in the Campeche Sound had a positive statistical association with hydrocarbons and heavy metals, while the parasite species and individuals infecting *S. papillosum* in the YS have a negative association with these compounds. Even when the YS is not free of hydrocarbons or heavy metals, their concentrations are apparently not high enough to affect the intermediate hosts (e.g. copepods, molluscs and shrimps) of metazoan parasites living in the benthic zone. Increasing levels of environmental contamination should lead to a decrease in parasite species and individuals, as has been reported for flounder (*Platichthys flesus*) [78] and Mayan sea catfish (*Ariopsis assimilis*) [79].

Vidal-Martínez et al. [3] suggested that the pattern of high numerical dominance in larval cestodes and digeneans of flatfish species (e.g. *S. gunteri*, *C. chittendeni* and *S. plagiusa*) captured in Campeche Sound may reflect a transition in the composition of the parasite community from contaminant-sensitive parasite species to contaminant-resistant species. This suggestion was based on the observation that the parasite data for these three flatfish species originated from a zone influenced by oil-extraction activities and that the community metrics of their parasite fauna were likely reflecting a highly-disturbed environment. These authors indicated that testing this hypothesis would require comparing their data with parasite communities of flatfishes in less-impacted environments in the Gulf of Mexico. In the present study, the parasite communities of *S. papillosum* were obtained from a less-impacted environment and were dominated numerically by larval cestodes and digeneans but harboured a large number of rare species. Therefore, parasite communities of flatfishes being dominated by larval stages appears to be a common pattern in both impacted and healthy environments in the Gulf of Mexico. The difference between impacted and healthy environments is due to the large number of rare parasite species of nematodes, acanthocephalans and arthropods present in the healthy environment (Table 2) [3, 7].

## Conclusions

In conclusion, the metazoan parasite infracommunities of *S. papillosum* presented high parasite biodiversity in the Yucatan Shelf, as seen from the high number of species and individuals compared to those reported for *S. gunteri* from the Campeche Sound. However, the region is influenced by seasonal oceanographic processes since the significant differences among subregions in parasite infracommunity metrics of *S. papillosum* apparently were due to the interruption of the Yucatan current during the north wind season. Additionally, the YS is not exempt of anthropogenic disturbance such as the fishing of top predators (i.e. sharks and rays). In fact, this study agrees with prior studies regarding the negative effects of fishing on the richness of parasites species [6, 8, 9, 73–75]. It is not known whether the parasite communities of *S. papillosum* and other flatfish species in this region will be resilient in the future, as an environmental catastrophe (e.g. an oil-spill) has not yet occurred. It remains to be seen what the future will hold for these highly-diverse parasite communities over the next few years if the oil-extraction plans in this region are implemented.

## Additional files

**Additional file 1: Table S1.** Environmental variables of sediment, water and organisms from sampling sites on the YS.

**Additional file 2.** Metazoan parasites of *Syacium papillosum*. Microphotographs and measurements.

## Data Availability

All data generated or analysed during this study are included in this published article and its additional files.

## Abbreviations

YS: Yucatan Platform
ED: explained deviance
