# Supplementary material for: Metazoan parasite infracommunities of the dusky flounder (Syacium papillosum) as bioindicators of environmental conditions in the continental shelf of the Yucatan Peninsula, Mexico
Source: Parasit Vectors. 2019 May 31;12:277. doi: 10.1186/s13071-019-3524-6 (PMC6545031; doi:10.1186/s13071-019-3524-6)
Supplement: Supplementary file 2 — Additional file 2. Metazoan parasites of Syacium papillosum. Microphotographs and measurements. [file 13071_2019_3524_MOESM2_ESM.pdf]

Metazoan parasites of *Syacium papillosum*. Photographic material and measurements (given in micrometers, otherwise mentioned).

The following photographs and measurements are presented to sustain our claim that we are dealing with different species of helminths, even for larval stages of digeneans. The material has been organized in phylogenetic order. In several cases measurements were not include because parasite specimens belong to new species that will be published elsewhere.

MONOGENEA

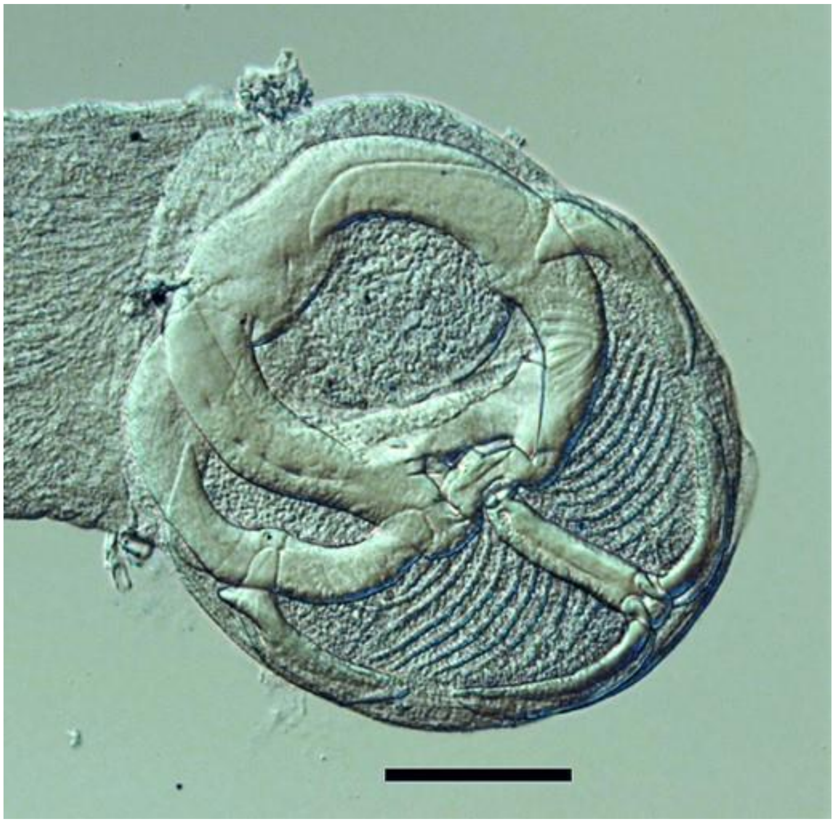

Figure 1. *Neoheterobotrium* n. sp. This is a new species for science and will be fully described elsewhere. Bar=100 micrometers.

DIGENEA

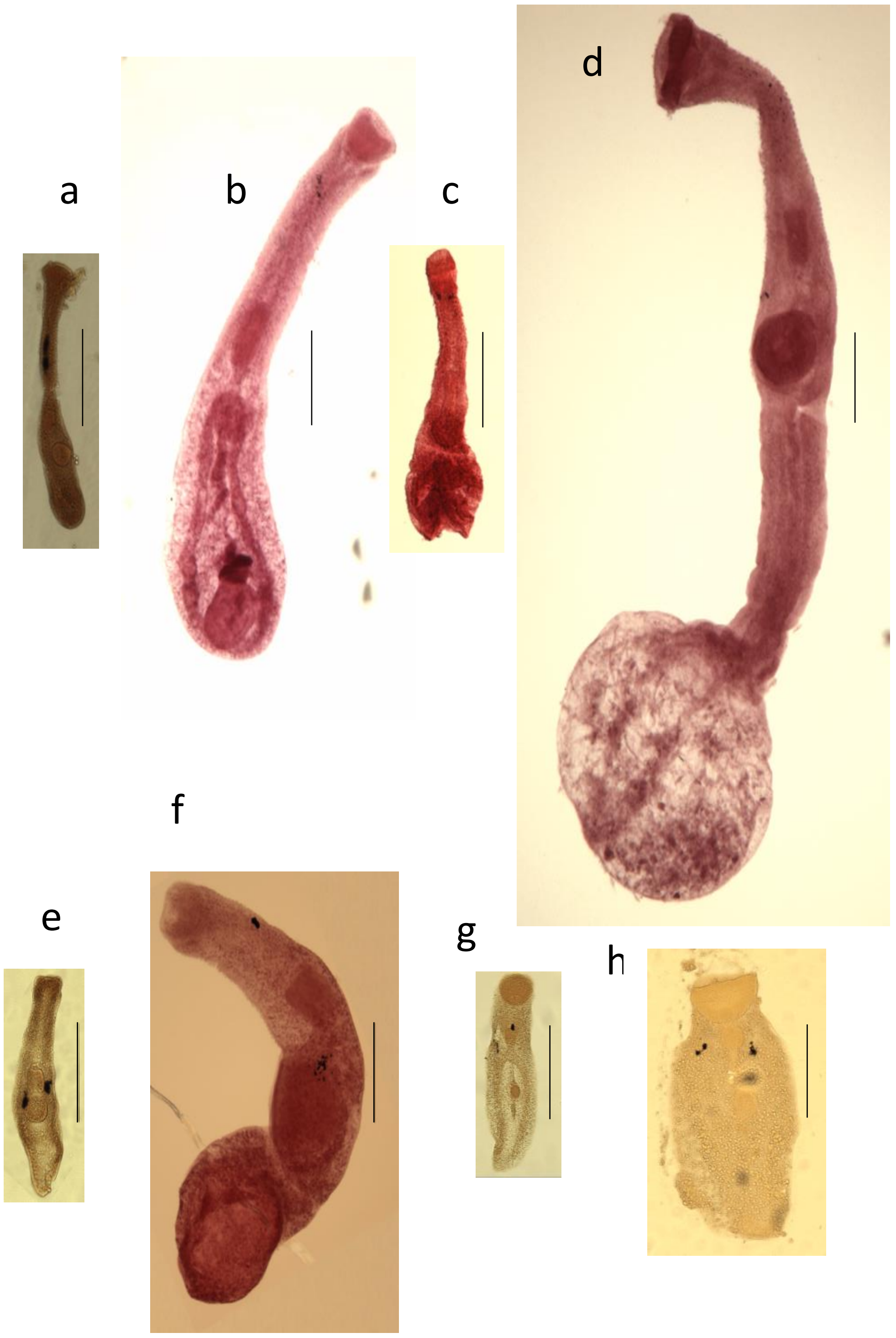

Figure 2. a) *Stephanostomum* sp. 1; b) *Stephanostomum* sp. 2; c) *Stephanostomum* sp. 3; d) *Stephanostomum* sp. 4; e) *Stephanostomum* sp. 5; f) *Stephanostomum* sp. 6; g) *Cryptogonimidae* gen sp. 1; h) *Cryptogonimidae* gen sp. 2. Bar=500 micrometers.

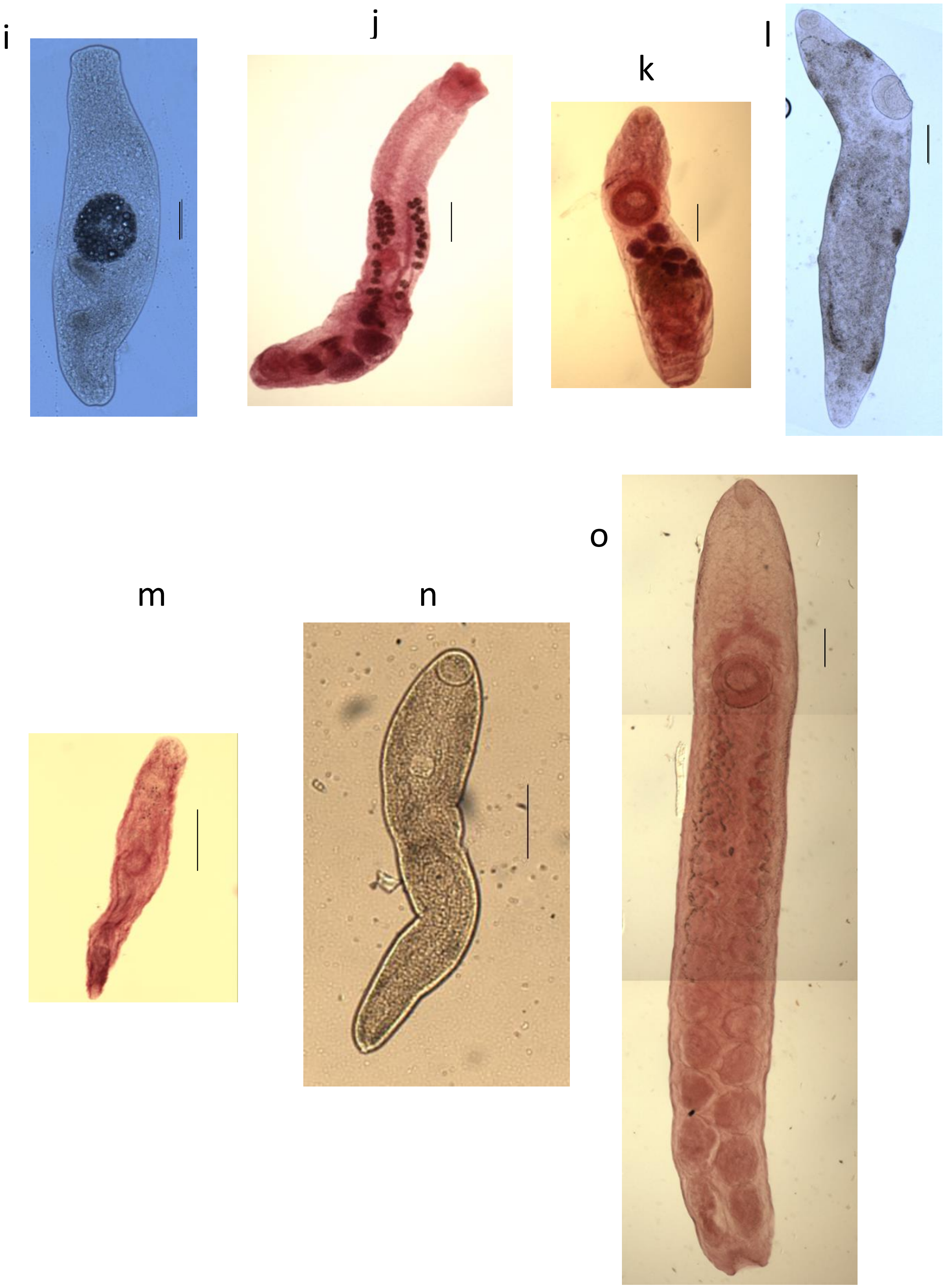

Figure 3. i) *Prosorhynchus* sp.; j) *Rhipidocotyle* sp.; k) *Lecithochirium floridense*; l) *Lecithochirium* sp.; m) *Lepocreadium* sp.; n) *Lepidapedon* sp.; o) Didymozoidae (Neotorticaecum-like). Bar=500 micrometers.

Table 1. Minimum-Maximum (mean) of *Stephanostomum* metacercariae found in *Syacium papillosum* from the Yucatan shelf, southern Mexico.

Stephanostomum		**sp. 1** Min-Max (Mean)	**sp. 2**	**sp.3**	**sp. 4**	**sp. 5**	**sp. 6**
Body	length	1587-1816 (1702)	1231-2579 (1850)	1265-1808 (1536)	1651.63-2705.56 (1986.62)	1209-1580 (1433)	2274-3310 (2792)
	width	219-256 (238)	228-463 (284)	297-571 (434)	268.03-669.43 (464.83)	331-421 (366)	672-676 (674)
Oral sucker	length	149-194 (171)	109-168 (137)	130-158 (144)	102.22-152.84 (129.92)	77-128 (95)	264-289 (276)
	width	123-192 (157)	113-167 (137)	194-230 (212)	130.11-205.68 (167.23)	119-233 (175)	334-370 (352)
Spines	Number	40	60-62 (61)	48	42	32	44
Ventral sucker	length	144-161 (152)	99-155 (127)	136-183 (160)	140.91-193.04 (166.89)	179-240 (201)	442-578 (510)
	width	131-162 (146)	95-153 (122)	168-211 (190)	116.28-180.04 (149.65)	149-237 (185)	481-515 (498)
Sucker radio	1:	0.86-0.94 (0.90)	0.76-1.05 (0.90)	0.78-1.21 (0.99)	1-1.10 (1.06)	1.35-1.51 (1.42)	1.48-1.72 (1.60)
	Distance*	1070-1115 (1093)	591-1362 (971)	839-968 (903.39)	647.77-941.86 (792.06)	700-1002 (866)	1280-1615 (1447)
	%	61.3-67 (64.4)	48.-55 (52)	53-66 (60)	31.1-45.5 (40.6)	57-66 (60)	49-56 (52)

*=distance from the Ventral sucker to the anterior end of the body; % = distance as % of body length

Table 2. Measurements of metacercariae of the family Cryptogonimidae and Bucephalidae from *S. papillosusm* form the Yucatan shelf.

		Cryptogonimidae gen sp.	*Cryptogonimidae gen* sp.2			*Prosorhynchus* sp.	*Rhipidocotyle* sp.
		Min-Max (Mean)					
Body	length	1124-1248 (1195)	789-1656 (1131)	Body	length	1472-2167 (1842)	3829
	width	349-486 (409)	483-976 (662)		width	340-572 (445)	551
Oral sucker	length	154.4-218.28 (192.76)	162-356 (231)	Rhynchus	length	90-336 (199)	354
	width	168-303 (242)	314-513 (391)		width	157-212 (177)	337
Ventral sucker	diameter	64-108 (75)	74-141 (100)	Mouth	diameter	57-86 (74)	160
Sucker radio	1	0.30-0.41 (0.34)	0.1-0.31 (0.22)		Distance*	825-1420 (1139)	1942
	distance*	561-704 (624)	317-732 (478)		%	56-65 (61)	50
	%	46-56 (52)	40-44 (42)				

*=distance of the Ventral sucker respect to the anterior end of the body; % = distance as % of body length

Table 3. Morphological measurements of adult trematodes found in *S. papillosum* from the Yucatan shelf.

		Lecithochirium floridense Min-Max (mean)	*Lecithochirium* sp.	*Lepocreadium* sp.	*Lepidapedon* sp.	*Neotorticaecum* sp.
Body	length	1769-2523 (2035)	2610	1301	2588	3575-4715 (4054)
	width	360-488 (419)	408	242	470	450-604 (526)
Oral sucker	length	107-137 (117)	108	104	241	105-152 (122)
	width	105-128 (114)	110	104	312	77-132 (104)
Ventral sucker	length	300-308 (304)	253	80	249	136-290 (234)
	width	301-309 (304)	217	79	241	241-352 (296)
Sucker radio	01:00	2.32-2.84 (2.65)	2.158	0.76	0.89	1.71-2.91 (2.41)
	Distance*	454-557 (493)	583	666	1104	918-1060 (1016)
	%	18-31 (25)	22	51	43	22-30 (25)

*=distance of the Ventral sucker respect to the anterior end of the body; % = distance as % of body length

CESTODA

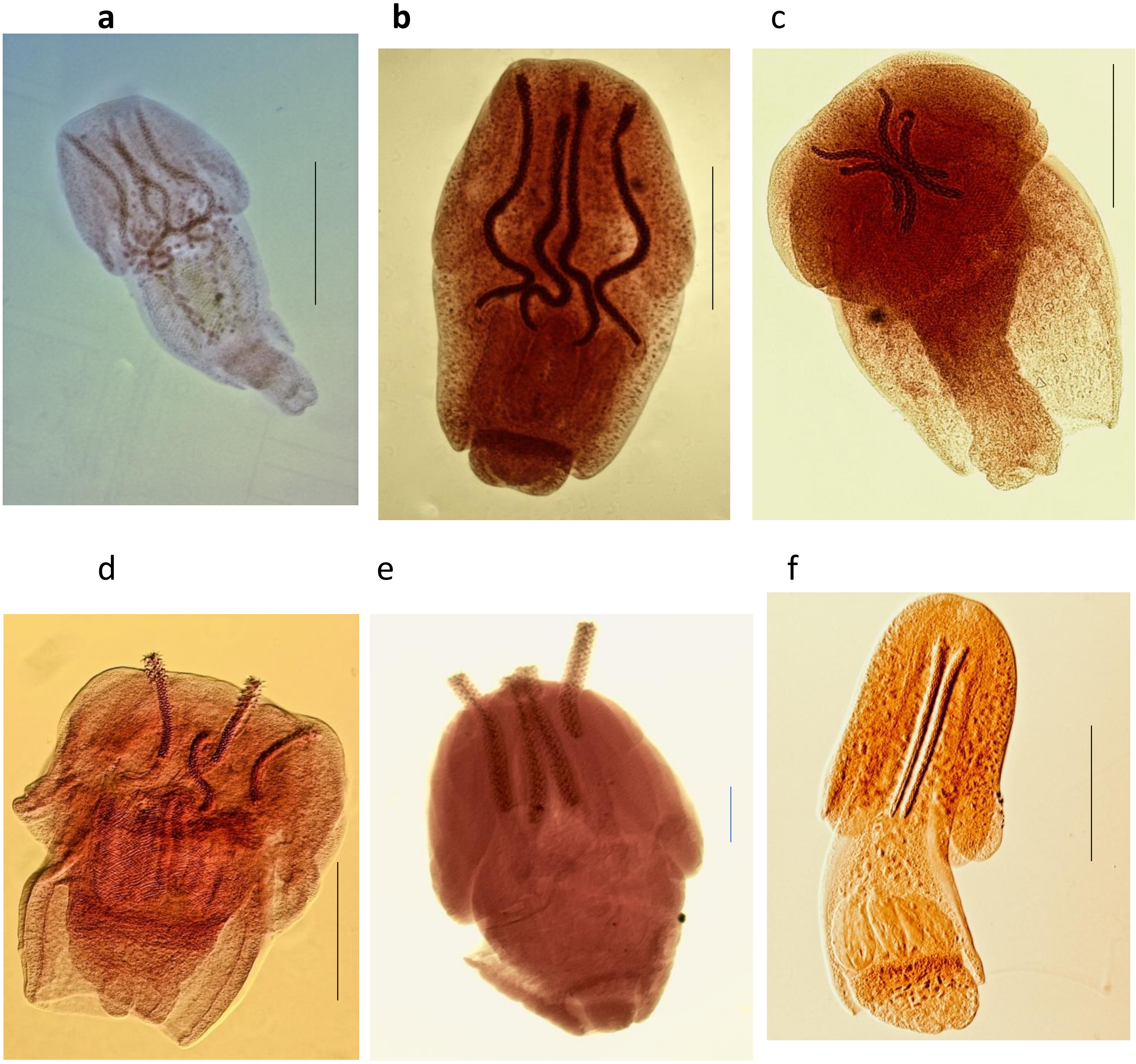

Figure 4. a) *Nybelinia* sp. 1; b) *Nybelinia* sp. 2; c) *Nybelinia* sp. 3; d) *Nybelinia* sp. 4; e) *Nybelinia* sp. 5; f) *Kotorella pronosoma*. Bar= 500 micrometers.

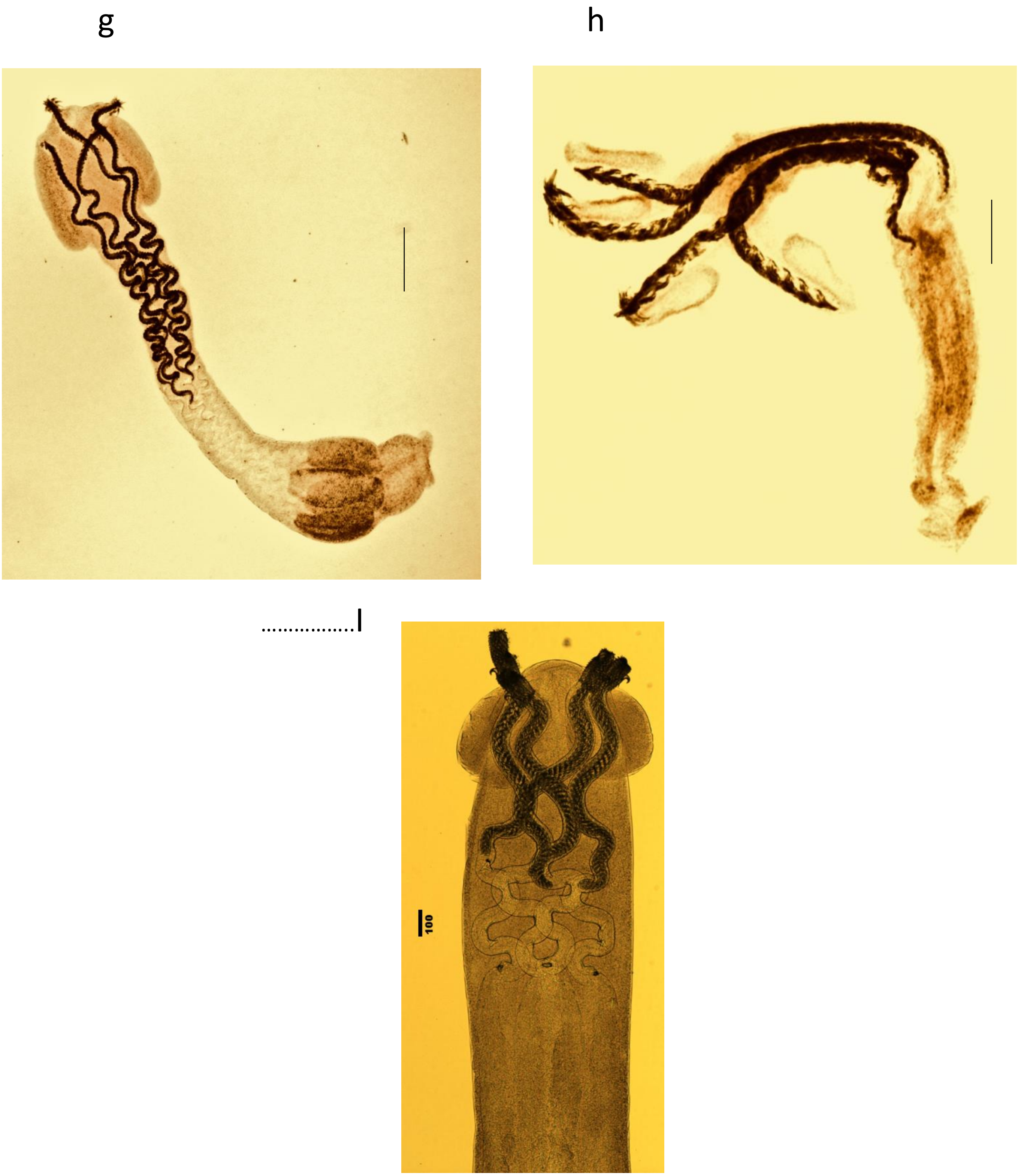

Figure 5. g) *Lacistorhynchus* sp.; h) *Pterobothrium* gen. sp.; i) *Oncomegas wageneri*. Bar= 500 micrometers.

Table 4. Measurements of cestodes in larval stage found in *S. papillosum* from the Yucatan shelf.

**Parasites** (no. Individuals)	**Scolex length (Max-Min) Mean**	**Pars bothridialis length**	**Pars vaginalis**	**Pars bulb length**	**Pars bulb width**	**Pedunculus scolesis**	**Tentacle length**
***Nybelinia* sp.1 (4)**	(699-839)<br>736	(289-382)<br>326.63	(290-397)<br>325	(179-200)<br>190.09	(65-74)<br>69	(566-681)<br>612	(372-399)<br>381
***Nybelinia* sp. 2 (2)**	(474-512)<br>493	(244-267)<br>256	(257-297)<br>277	(115-129)<br>122	(44-58)<br>51	(414-465)<br>439	(323-381)<br>352
***Nybelinia* sp. 3 (7)**	(501-803)<br>608	(213-367)<br>268	(192-298)<br>247	(93-168)<br>116	(36-82)<br>52	(411-755)<br>550	(108-249)<br>162
***Nybelinia* sp. 4 (6)**	(645-814)<br>736	(312-423)<br>362	(151-229)<br>185	(179-241)<br>225	(71-106)<br>83	(660-795)<br>733	(138-234)<br>200
***Nybelinia* sp. 5 (1)**	501	321	274	78	29	489	206
***Kotorella promosoma* (10)**	(607-1065)<br>859	(305.05-386)<br>353	(380-488)<br>432	(101-140)<br>114	(34-64)<br>46	(527-668)<br>642	(251-276)<br>261
***Lacistorhynchus* sp. (2)**	(716-758)<br>737	(83.53-85)<br>85	(287-292)<br>290	(62-64)<br>63	(19-25)<br>22	(344-352)<br>348	(285-302)<br>293
**Pterobothrium sp. (1)**	607	72	383	_	_	_	316

NEMATODA

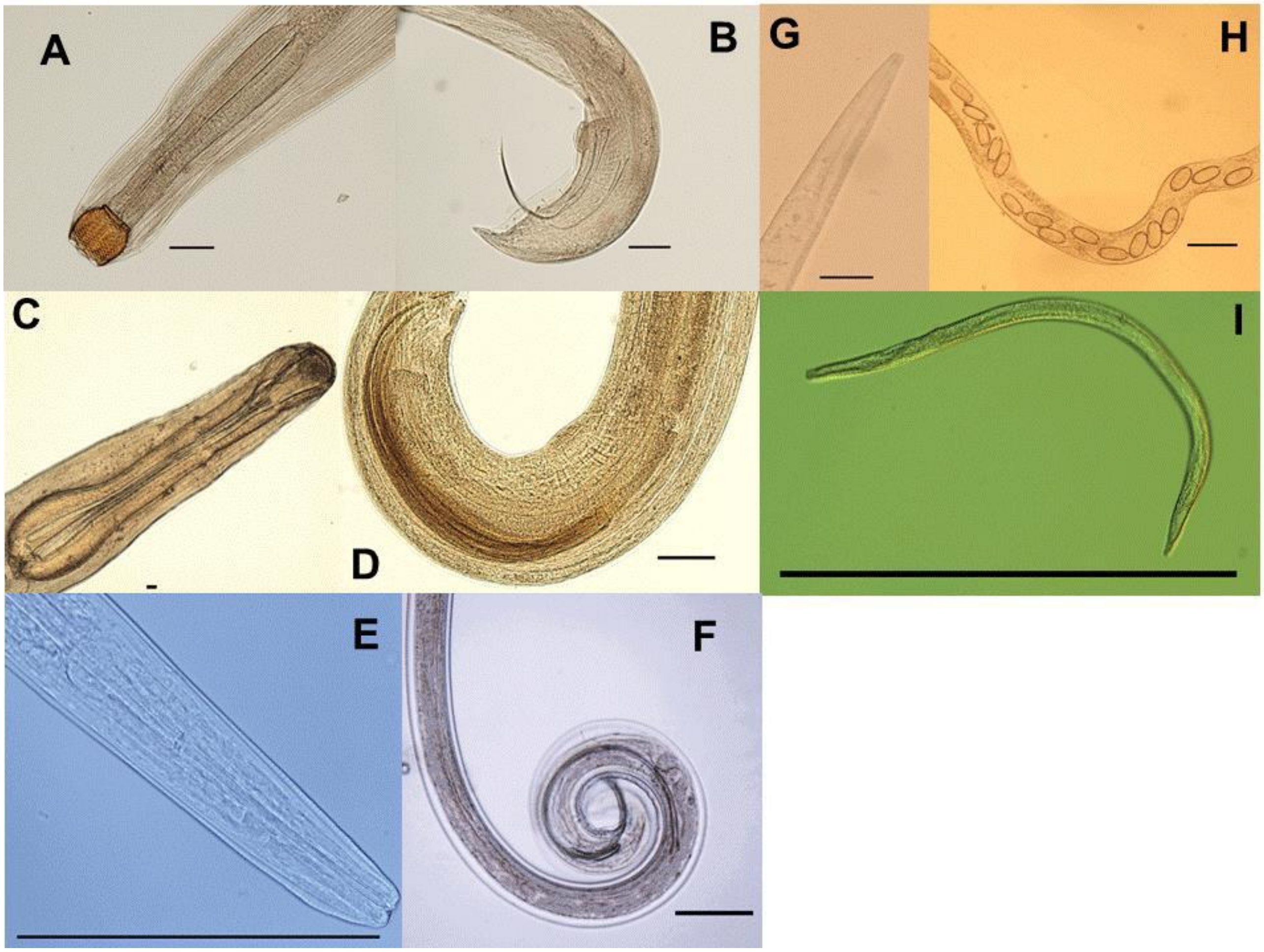

Figure 6. *Spirocamallanus chetumalensis*, A) anterior end, B) posterior end; *Cucullanus* sp., C) anterior end, D) posterior end; *Johnstonmawsonia* sp. E) anterior end (Scale bar: 50 µm), F) posterior end; Capillariidae gen. sp. G) anterior end, H) posterior end; I) *Anisakis* sp. (Scale bar: 100 µm).

ARTHROPODA

a b

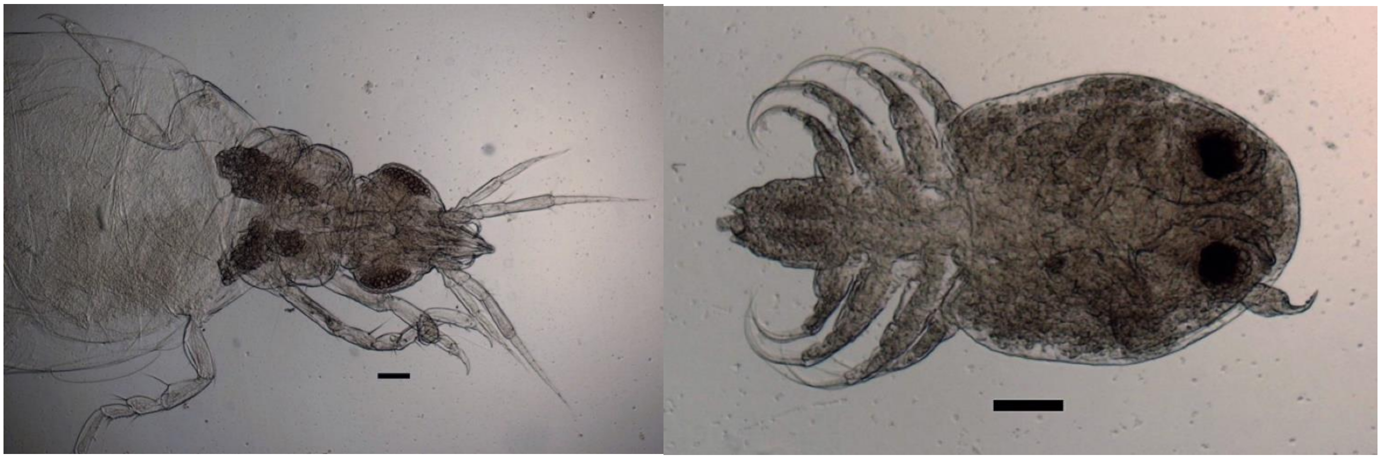

Figure 7. a. *Gnathia* sp. (scale bar= 100 micrometers); b. *Argulus* sp. (scale bar= 100 micrometers).
